# Digestible indispensable amino acid scores of animal and plant ingredients potentially used in dog diet formulation: how this protein quality metric is affected by ingredient characteristics and reference amino acid profile

**DOI:** 10.1093/jas/skac279

**Published:** 2022-08-27

**Authors:** James R Templeman, Anna K Shoveller

**Affiliations:** Department of Animal Biosciences, University of Guelph, Guelph, Ontario N1G 2W1, Canada; Department of Animal Biosciences, University of Guelph, Guelph, Ontario N1G 2W1, Canada

**Keywords:** amino acids, digestible indispensable amino acid score, pet food, protein quality

## Abstract

The ability of a diet or an ingredient to satisfy the indispensable amino acid (**IAA**) requirements of an individual is a reflection of protein quality (**PQ**). The concept of PQ is gaining recognition in the pet food industry as a way to identify candidate ingredients for diet formulation. The objective of this report was to use IAA digestibility data from swine and cecectomized rooster assays to generate digestible IAA scores (defined herein as **DIAAS**-like values) to predict the PQ of ingredients used in dog diets. However, as PQ equation development relies on a reference IAA profile, which is intended to be based on the physiological requirements of a specific population, we sought to generate DIAAS-like values using IAA requirements established by the National Research Council (**NRC**) as well as practical IAA recommendations presented by the Association of American Feed Control Officials (**AAFCO**) and European Pet Food Industry Federation (**FEDIAF**), to assess how these profiles may affect PQ. In total, 30 animal (75 unique inputs) and 27 plant ingredients (94 unique inputs) satisfied all inclusion criteria to be used in the final data set. Ingredients were initially categorized as animal or plant, and further categorized based on AAFCO Official Common and Usual Names and Definitions of Feed Ingredients to allow for additional, more distinct comparisons to be made. Data were analyzed using PROC GLIMMIX in SAS, with ingredient reference as a random effect, and ingredient category, regulatory body, and life stage as fixed effects. As expected, differences were observed in DIAAS-like values for nearly all ingredients and ingredient categories when determined using NRC, AAFCO, or FEDIAF IAA requirements or recommendations as the reference pattern. Moreover, applying reference patterns based on NRC adult maintenance IAA requirements consistently produced the lowest DIAAS-like values. Ultimately, while future studies assessing PQ should utilize NRC minimal requirements, individual ingredient and ingredient category differences in DIAAS-like values when using AAFCO and FEDIAF recommendations underpin the different regulatory approaches to establishing dietary nutrient recommendations that exist globally and support the need for harmonization of dietary recommendations.

## Teaser

Protein quality (**PQ**) of a variety of ingredients potentially used in commercial dog diet formulation was predicted using indispensable amino acid (**IAA**) profiles based on estimated physiological requirements as well as practical recommendations from various pet food regulatory bodies. The selection of IAA reference patterns based either on physiological requirements or practical dietary recommendations greatly affected the predicted PQ of the ingredients, ultimately supporting a need for optimization of PQ equations used in the pet food industry so as to align with those used in the human food industry.

## Introduction

Dogs, like other mammals, do not have a requirement for protein per se, but rather have a requirement for indispensable amino acids (**IAA**) and sufficient dispensable amino acids (**AA**) that a dietary source of protein is comprised of. These IAA cannot be endogenously synthesized by the dog, and thus must be supplied in the diet to support protein synthesis as well as a variety of secondary metabolic functions. The ability of a diet (e.g., a mixture of protein sources) or an individual protein-containing ingredient to satisfy the IAA requirements of an individual is a reflection of protein quality (**PQ**; Nosworthy and House, 2017). A concept known as AA bioavailability is fundamental to the determination of PQ and is defined as the proportion of AA liberated from a protein source and absorbed in a metabolically available form for body protein maintenance and/or growth (Batterham, 1992; Stein et al., 2007a). However, to holistically evaluate the nutritional value of a diet or protein-containing ingredient, IAA bioavailability must be considered in combination with the concentration of IAA present in that diet or ingredient (Prolla et al., 2013). For any protein source, the IAA present in the lowest concentration relative to the subject’s biological requirements is defined as the limiting AA (**LAA**), which ultimately determines the rate of metabolic utilization of all other IAA present, and thus is directly related to the assessment of PQ (Fuller and Reeds, 1998).

In human nutrition, the concept of PQ is well recognized and widely applied to facilitate guidance on the extensive range of food items humans consume. As such, the Food and Agriculture Organization (**FAO**) has developed a criterion to collect and compile measures of PQ, which include mathematical equations such as the protein digestibility-corrected AA score (**PDCAAS**) and the digestible indispensable AA score (**DIAAS**). These equations allow for PQ of a protein-containing food or ingredient to be calculated using existing data in the literature on physiological IAA requirements of a reference population as well as the IAA content and true total tract protein digestibility (for the PDCAAS equation) or true ileal AA digestibility (for the DIAAS equation) of that ingredient (FAO, 2013). Indeed, it is important to note that measures of AA digestibility are used as a proxy for AA bioavailability. Furthermore, it is essential to recognize that true total tract protein digestibility does not account for differences in individual AA digestibility. Due to the inherent issues related to the use of total tract protein digestibility and potential for overestimation of AA bioavailability by using protein digestibility as an approximation of all AA bioavailability (Darragh et al., 1994; Murray et al., 1997; Stein et al., 2007a; Columbus et al., 2014), the DIAAS method (using ileal AA digestibility data) is considered to be a more accurate assessment of PQ (FAO, 2013).

In the pet food industry, the concept of PQ is gaining recognition as a way in which to identify and select ingredients for diet formulation. Though, while it is recognized that the bioavailability of AA should be estimated using true ileal AA digestibility data, due to the ethical and economical restraints surrounding the measurement of ileal digestibility in dogs, there remains a dearth of literature to support the generation of PQ equations such as DIAAS for dogs. Swine are acknowledged as excellent models for the digestive capacity and metabolism of AA in humans (Moughan and Rowan, 1989; Rowan et al., 1994; Deglaire et al., 2009), and in fact, data generated from swine ileal AA digestibility studies are used to supplement the published data available in humans for the generation of the DIAAS PQ equation. However, the pet food industry remains largely reliant on canine total tract protein digestibility data as well as estimations of dietary protein and AA bioavailability presented by the National Research Council (Nutrient Requirements for Dogs and Cats; NRC, 2006) as well as the North American (Association of American Feed Control Officials; AAFCO, 2016) and European (European Pet Food Industry Federation; FEDIAF, 2018) pet food regulatory bodies, even though these estimations have been criticized for a potential lack of scientific credibility and accuracy (Morris and Rogers, 1994; Hendriks et al., 2015). As such, the primary objective of this report was to use IAA digestibility data generated from comparative species that share similar digestive anatomy and protein/AA metabolism with the domestic dog, specifically swine (Harrison et al., 1990) and the cecectomized rooster (Johnson et al., 1998), to generate DIAAS-like values to predict the PQ of protein-containing ingredients used by the pet food industry. To accomplish this, the authors assessed the applicable literature related to swine ileal AA digestibility and cecectomized rooster model AA digestibility data for plant and animal protein-containing ingredients used for dog diet formulation.

It should be noted, though, that since the DIAAS equation requires the IAA content and ileal IAA digestibility coefficients for a test diet or test ingredient along with the IAA requirements of a reference population to compare to (FAO, 2013), the PQ (DIAAS) of an ingredient may be affected by the reference population selected. Previously, as per a joint FAO/World Health Organization/United Nations University report (FAO/WHO/UNU, 1985), United States regulations stipulated that IAA requirements of preschool children aged 2 to 5 be used as a reference population for the evaluation of PQ for all age groups, aside from infants. However, following a more recent evaluation of these recommendations, the FAO now recommends that reference patterns based on IAA requirements of 0.5-yr-old infants and 3 to 10-yr-old children be used to calculate PQ metrics, such as DIAAS, for young children (0.5 to 3 yr of age) and older children/adolescents/adults (3+ yr of age), respectively (FAO, 2013). As dogs are typically fed a single complete diet that has been formulated to meet or exceed the IAA requirements of the physiological stage the diet is intended for (gestation/lactation/growth or adult maintenance), it is logical that steps are taken to ensure that estimations of PQ for dogs are made using life stage-specific IAA requirements as a reference pattern.

Nevertheless, for the pet food industry to appropriately adopt PQ metrics such as DIAAS, it is essential that the same procedures are followed when utilizing these equations as are in the human food industry (FAO, 2013). Specifically, when calculating DIAAS for individual protein sources, the most appropriate IAA reference patterns to apply are those based on physiological requirement estimates (e.g., children aged 3 to 10 yr; FAO, 2013). In the case of dogs, this would refer to IAA reference patterns based on minimal requirement (**MR**) estimates established by the NRC (2006) for adult dogs as well as for growing puppies. However, in recent years, a number of reports have been published wherein DIAAS for protein ingredients potentially used in commercial dog diet formulation have been calculated using reference patterns based on physiological IAA requirements from the NRC as well as practical dietary IAA recommendations from pet food regulatory bodies, such as AAFCO (Oba et al., 2019; Do et al., 2020; Reilly et al., 2020a, 2021; Gomez et al., 2021). It should be noted, though, that for each of these aforementioned reports, DIAAS values generated were referred to as “DIAAS-like” values, and the DIAAS equations utilized were adapted from the equation defined by the FAO (2013) so as to capture the fact that the IAA reference pattern utilized was generated using the “minimum protein recommendation” (Oba et al., 2019) rather than a “reference protein” (FAO, 2013).

Therefore, the secondary objective was to similarly generate DIAAS-like values for the ingredients of interest based on each reference population established by the NRC (adult maintenance, growing puppies 4 to 14 wk old, growing puppies 14 wk and older), as well as those defined by AAFCO (adult maintenance, growth and reproduction) and FEDIAF (adult maintenance, early growth, and late growth). This was undertaken to establish how choice of regulatory body and/or life stage may affect the interpretation of an ingredient’s PQ while also offering the opportunity to provide quantitative data that DIAAS-like values generated using reference patterns based on NRC physiological requirement estimates will most closely reflect the DIAAS PQ metric recommended by the FAO (2013). We hypothesize that given the differences in crude protein (**CP**) and IAA requirements/recommendations from NRC, AAFCO, and FEDIAF, applying IAA reference patterns based on adult maintenance specifications will result in lower DIAAS-like values compared with those calculated using growth specifications, and that DIAAS-like values calculated using reference patterns based on NRC will be the lowest, followed by those calculated using AAFCO and then FEDIAF. Ultimately, the data generated will help to facilitate guidance on the formulation of dog diets with the intention of more precisely meeting the protein and IAA requirements or recommendations of the intended population by allowing for quantitative selection of protein-containing ingredients based on PQ predictions.

## Materials and Methods

### Data collection

In order to be captured in the final data set, all ingredients had to fulfill the following requirements: 1) have accessible literature describing moisture or dry matter (**DM**) content, CP content, and IAA profile (including Tyr and Cys for assessment of total aromatic AA (**AAA**) and total sulfur AA (**SAA**), respectively) so as to present IAA data with units of mg/g CP; 2) have accessible literature describing the standardized ileal digestibility coefficients from swine or digestibility coefficients from cecectomized roosters for each IAA; 3) have a CP content of ≥8% on a DM basis; and 4) be considered as a source of animal or plant-derived protein, where they can then be further classified/categorized based on the Official Common and Usual Names and Definitions of Feed Ingredients as established by AAFCO (2016).

It is acknowledged that while the IAA digestibility coefficients included in this report from swine are based on standardized ileal digestibility measurements (Stein et al., 2007a), the cecectomized rooster assay data are based on true digestibility values determined using methodologies described by Sibbald (1979) and do not eliminate the minor contributions of colonic digestion, fermentation, and metabolism in the rooster. As such, the approach of determining true AA digestibility with the cecectomized rooster model differs from that determining true “ileal” digestibility commonly applied in swine and murine models (Stein et al., 2007b). These methodologies may yield marginally different results, yet both assays are considered to be comparable in nature to the ileally cannulated dog (Harrison et al., 1990; Johnson et al., 1998).

### Ingredient classification

In total, 30 animal (75 unique inputs from literature) and 27 plant ingredients (94 unique inputs from literature) satisfied all inclusion criteria and were captured in the final data set. For clarity, “casein” would be considered a single animal ingredient with three unique inputs, as IAA data were obtained from three unique sources (NRC, 2012; Rojas and Stein, 2012; CVB Feed Table, 2016). After being initially identified as “animal” or “plant,” all ingredients were then further categorized based on “collective,” “broad,” and “specific” AAFCO classifications, as defined using AAFCO (2016) Official Common and Usual Names and Definitions of Feed Ingredients (Tables 1 and 2 for animal and plant ingredients, respectively). It should be noted that, when necessary, common names were utilized when “specifically” defining certain ingredients (e.g., “dried pea”; Table 2). For a more detailed breakdown of the ingredient classification, refer to Supplementary Material S1.

**Table 1.** Animal ingredient (30 ingredients, 75 unique inputs) specifications and AAFCO classifications

Ingredient detail				AAFCO Classification		
Variety	Sort	Type	*n* ^1^	Collective	Broad	Specific
Cattle	Beef	Lung meal	1	Animal protein product	Animal product	Animal by-product meal
Cattle	Beef	Blood meal	1	Animal protein product	Animal product	Blood meal
Cattle	Beef	Loin	1	Animal protein product	Animal product	Meat
Cattle	Beef	Ground	1	Animal protein product	Animal product	Meat
Cattle	Milk	Casein	3	Animal protein product	Milk product	Casein
Cattle	Milk	Whole	2	Animal protein product	Milk product	Dried milk
Cattle	Milk	Skimmed	3	Animal protein product	Milk product	Dried skimmed milk
Cattle	Milk	Whey protein concentrate	3	Animal protein product	Milk product	Dried whey concentrate
Cattle	Milk	Whey	3	Animal protein product	Milk product	Dried whey
Fish	N/A^2^	Meal	4	Animal protein product	Marine product	Fish meal
Fish	Pollock	Meat	1	Animal protein product	Marine product	Meat
Fish	Salmon	Meat	1	Animal protein product	Marine product	Meat
Poultry	Chicken	Meat	3	Animal protein product	Animal product	Meat
Poultry	Chicken	Meal	3	Animal protein product	Animal product	Poultry meal
Poultry	Chicken	Neck meal	1	Animal protein product	Animal product	Poultry by-product meal
Poultry	Chicken	Chicken by-product meal	1	Animal protein product	Animal product	Poultry by-product meal
Poultry	Chicken	Breast	1	Animal protein product	Animal product	Meat
Poultry	Egg	Whole	2	Animal protein product	Animal product	Egg product
Poultry	N/A	Poultry by-product meal	4	Animal protein product	Animal product	Poultry by-product meal
Poultry	N/A	Meal	2	Animal protein product	Animal product	Poultry meal
Poultry	N/A	Feather meal	8	Animal protein product	Animal product	Hydrolyzed poultry feathers
Swine	Pork	Liver meal	1	Animal protein product	Animal product	Animal by-product meal
Swine	Pork	Lung meal	1	Animal protein product	Animal product	Animal by-product meal
Swine	Pork	Loin	1	Animal protein product	Animal product	Meat
Sheep	Lamb	Meal	1	Animal protein product	Animal product	Meat meal
Sheep	Lamb	Lung meal	1	Animal protein product	Animal product	Animal by-product meal
N/A	N/A	Meat and bone meal	8	Animal protein product	Animal product	Meat and bone meal
N/A	N/A	Bone meal	1	Animal protein product	Animal product	Animal by-product meal
N/A	N/A	Meat meal	5	Animal protein product	Animal product	Meat meal
N/A	N/A	Blood meal	6	Animal protein product	Animal product	Blood meal

Number of unique inputs per ingredient; see footnotes of Table 4 for literatures source of each unique input.

Data not available due to ingredient variety or sort being unknown.

**Table 2.** Plant ingredient (27 ingredients, 94 unique inputs) specifications and AAFCO classifications

Ingredient detail				AAFCO classification		
Variety	Sort	Type	*n* ^1^	Collective	Broad	Specific
Grain	Barley	Whole	4	Grain product	Barley product	Barley grain
Grain	Canola	Meal	2	Plant protein product	Other oilseed product	Canola meal
Grain	Cottonseed	Meal	2	Plant protein product	Cottonseed product	Cottonseed meal
Grain	Corn	Germ meal	3	Plant protein product	Maize product	Corn germ meal
Grain	Corn	Gluten meal	5	Plant protein product	Maize product	Corn gluten meal
Grain	Corn	Whole	6	Grain product	Maize product	Corn grain
Grain	Corn	DDGS^2^	2	Processed grain by product	Distillers product	DDGS
Grain	Flaxseed	Meal	3	Plant protein product	Other oilseed product	Linseed meal
Grain	Oat	Whole	3	Grain product	Oat product	Oat grain
Grain	Oat	Groats	3	Processed grain by product	Oat product	Oat groats
Grain	Peanut	Meal	2	Plant protein product	Other oilseed product	Peanut meal
Grain	Rice	Bran	2	Processed grain by product	Rice product	Rice bran
Grain	Rice	Whole	4	Grain product	Rice product	Rice grain
Grain	Rye	Whole	4	Grain product	Rye product	Rye grain
Grain	Sorghum	Whole	4	Grain product	Grain sorghum	Sorghum grain
Grain	Sunflower	Meal	2	Plant protein product	Other oilseed product	Sunflower meal
Grain	Wheat	Bran	4	Processed grain by product	Wheat product	Wheat bran
Grain	Wheat	Middlings	3	Processed grain by product	Wheat product	Wheat middlings
Grain	Wheat	DDGS	2	Processed grain by product	Distillers product	DDGS
Grain	Wheat	Whole	5	Grain product	Wheat product	Wheat grain
Legume	Soybean	Meal	5	Plant protein product	Soybean product	Soybean meal
Legume	Soybean	Protein concentrate	3	Plant protein product	Soybean product	Soy protein concentrate
Legume	Lupin bean	Whole	1	Plant protein product	Miscellaneous product	Dried bean
Legume	Fava bean	Whole	5	Plant protein product	Miscellaneous product	Dried bean
Legume	Pea	Whole	7	Plant protein product	Miscellaneous product	Dried pea
Tuber	Potato	Protein concentrate	5	Plant protein product	Miscellaneous product	Potato protein
Yeast	Yeast	Brewers	3	Plant protein product	Yeast product	Brewers dried yeast

Number of unique inputs per ingredient; see footnotes of Table 5 for literatures source of each unique input.

Distillers dried grain with solubles.

### PQ calculations—reference IAA patterns

Nutritional data (e.g., CP content, IAA profiles, and IAA digestibility) were gathered for the purpose of generating ingredient-specific DIAAS-like values. This metric necessitates a “reference pattern” to compare the digestible IAA content of each ingredient to, and as such, DIAAS-like values were determined using estimations of physiological IAA requirements presented by the NRC (2006) as well as the practical dietary IAA recommendations presented by the North American (AAFCO, 2016) and European (FEDIAF, 2018) pet food regulatory bodies.

Moreover, given that these organizations have presented estimated IAA requirements (or practical dietary IAA recommendations) for a variety of canine life stages, discrete reference IAA patterns were compiled for each of these, including adult maintenance (**AM**; NRC, 2006; AAFCO, 2016; FEDIAF, 2018); early growth (**EG**; 4 to 14 wk of age, NRC, 2006; less than 14 wk of age, FEDIAF, 2018); late growth (**LG**; greater than 14 wk of age, NRC, 2006; FEDIAF, 2018), and; growth and reproduction (GR; AAFCO, 2016). These IAA requirements/recommendations (presented on a g per 100 g DM basis) as well as the IAA reference patterns (transformed to and presented on a mg/g CP basis) are outlined in Table 3 and the latter was used for the determination of DIAAS-like values.

**Table 3.** Crude protein content and indispensable amino acid requirements or recommendations as published by NRC, AAFCO, or FEDIAF for each discrete life stage presented on a percent of dry matter basis and the respective indispensable amino acid reference patterns presented on a mg/g crude protein basis

	NRC												AAFCO				FEDIAF					
	AM^1^				EG				LG				AM		GR		AM (110 kcal/kg^0.75^)		EG		LG	
	MR^2^ %	RA DM^3^	MR mg/g	RA CP^4^	MR %	RA DM	MR mg/g	RA CP	MR %	RA DM	MR mg/g	RA CP										
													% DM	mg/g CP	% DM	mg/g CP	% DM	mg/g CP	% DM	mg/g CP	% DM	mg/g CP
CP^5^	8.00	10.00	-	-	18.00	22.50	-	-	14.00	17.50	-	-	18.00	-	22.50	-	18.00	-	25.00	-	20.00	-
Arg	0.28	0.35	**35.00**	35.00	0.63	0.79	**35.00**	35.11	0.53	0.66	**37.86**	37.71	0.51	**28.33**	1.00	**44.44**	0.52	**28.89**	0.82	**32.80**	0.74	**37.00**
His	0.15	0.19	**18.75**	19.00	0.31	0.39	**17.22**	17.33	0.20	0.25	**14.29**	14.29	0.19	**10.56**	0.44	**19.56**	0.23	**12.78**	0.39	**15.60**	0.25	**12.50**
Ile	0.30	0.38	**37.50**	38.00	0.52	0.65	**28.89**	28.89	0.40	0.50	**28.57**	28.57	0.38	**21.11**	0.71	**31.56**	0.46	**25.56**	0.65	**26.00**	0.50	**25.00**
Leu	0.54	0.68	**67.50**	68.00	1.03	1.29	**57.22**	57.33	0.65	0.82	**46.43**	46.86	0.68	**37.78**	1.29	**57.33**	0.82	**45.56**	1.29	**51.60**	0.80	**40.00**
Lys	0.28	0.35	**35.00**	35.00	0.70	0.88	**38.89**	39.11	0.56	0.70	**40.00**	40.00	0.63	**35.00**	0.90	**40.00**	0.42	**23.33**	0.88	**35.20**	0.70	**35.00**
Phe^6^	0.36	0.45	**45.00**	45.00	0.52	0.65	**28.89**	28.89	0.40	0.50	**28.57**	28.57	0.45	**25.00**	0.83	**36.89**	0.54	**30.00**	0.65	**26.00**	0.50	**25.00**
AAA	0.59	0.74	**73.75**	74.00	1.04	1.30	**57.78**	57.78	0.80	1.00	**57.14**	57.14	0.74	**41.11**	1.30	**57.78**	0.89	**49.44**	1.30	**52.00**	1.00	**50.00**
Met	0.26	0.33	**32.50**	33.00	0.28	0.35	**15.56**	15.56	0.21	0.26	**15.00**	14.86	0.33	**18.33**	0.35	**15.56**	0.40	**22.22**	0.35	**14.00**	0.26	**13.00**
SAA	0.52	0.65	**65.00**	65.00	0.56	0.70	**31.11**	31.11	0.42	0.53	**30.00**	30.29	0.65	**36.11**	0.70	**31.11**	0.76	**42.22**	0.70	**28.00**	0.53	**26.50**
Thr	0.34	0.43	**42.50**	43.00	0.65	0.81	**36.11**	36.00	0.50	0.63	**35.71**	36.00	0.48	**26.67**	1.04	**46.22**	0.52	**28.89**	0.81	**32.40**	0.64	**32.00**
Trp	0.11	0.14	**13.75**	14.00	0.18	0.23	**10.00**	10.22	0.14	0.18	**10.00**	10.29	0.16	**8.89**	0.20	**8.89**	0.17	**9.44**	0.23	**9.20**	0.21	**10.50**
Val	0.39	0.49	**48.75**	49.00	0.54	0.68	**30.00**	30.22	0.45	0.56	**32.14**	32.00	0.49	**27.22**	0.68	**30.22**	0.59	**32.78**	0.68	**27.20**	0.56	**28.00**

AM, adult maintenance (using 110 kcal/kg^0.75^ for FEDIAF); EG, early growth (NRC, 4 to 14 wk of age; FEDIAF, less than 14 wk of age); LG, late growth (NRC and FEDAF, greater than 14 wk of age), GR, growth and reproduction (AAFCO only).

MR, minimal requirement; RA, recommended allowance.

Crude protein content and indispensable amino acid requirements presented on a % dry matter basis.

Indispensable amino acid reference patterns presented on a mg/g crude protein basis (used for DIAAS-like value calculations).

CP, crude protein; AAA, aromatic amino acids (Phe + Tyr); SAA, sulfur amino acids (Met + Cys).

In this report, reference values for Met and Phe were calculated as well as reference values for total SAA and AAA, while the FAO (2013) only calculates reference values for the latter.

Bolded used for the calculation of DIAAS-like values.

However, as DIAAS is intended to be established by comparing the physiological requirement of an IAA to the bioavailability (digestibility, in this case) of that IAA in a particular dietary protein source (FAO, 2013), the model IAA reference patterns to be utilized for dogs are those based on MR estimates by the NRC (2006) for adult dogs and growing puppies. With regard to the dietary recommended allowance (**RA**) estimates proposed by the NRC (2006), these are determined by multiplying the MR estimates by a factor of 1.25, which accounts for an average protein/IAA digestibility in a commercial diet of 80%. As this factor is used to transform the MR estimates for both IAA and CP (e.g., adult CP MR of 8%, adult CP RA of 10%; NRC, 2006), the net result is that the MR and RA estimates of the NRC (2006) will yield the same IAA reference patterns (see Table 3) and thus the same DIAAS.

For AAFCO and FEDIAF, recommendations for minimum levels at which IAA should be present in practical, commercial pet foods for dogs are essentially the RA estimates established by the NRC (e.g., account for average digestibility of 80%), aside from select instances where additional scaling factors have been implemented. For example, the FEDIAF adult maintenance IAA recommendations are NRC RA estimates increased by 20% to account for lower estimated energy requirements of household dogs compared to the energy intake assumed by NRC (FEDIAF, 2018). However, while CP recommendations from AAFCO and FEDIAF do also account for digestibility of the dietary protein sources, they are determined differently from IAA. For example, both regulatory bodies adopted a CP recommendation for adult dogs of 18%, which is based on the estimated MR of 8% presented by the NRC scaled up to account for requirements of older/senior dogs (upwards of 50%), lower energy intakes for household dogs (difference of 20%), as well as the average/assumed CP digestibility in a commercial diet of 80% (AAFCO, 2016; FEDIAF, 2018).

In brief, what this suggests is that, yes, using reference IAA patterns based on these AAFCO or FEDIAF recommendations will generate different DIAAS-like values than those determined using NRC MR estimates; however, these data must be interpreted with caution as these reference patterns no longer represent the physiological requirements of the dog, but rather account for assumed digestibility of the protein source. That being acknowledged, with the propensity of the pet food industry to rely on the recommendations presented by AAFCO and FEDIAF for commercial formulation purposes, and with the recent literature assessing PQ of ingredients intended for dog diet formulation using reference IAA patterns based on these regulatory bodies (Oba et al., 2019; Do et al., 2020; Reilly et al., 2020a, 2020b, 2021; Gomez et al., 2021), it may be valuable to assess the extent to which using these reference patterns may affect the PQ interpretation.

### PQ calculations—DIAAS-like values

DIAAS-like values were calculated using equations adapted from the FAO (2013):


$$\begin{array}{ll} & \mathrm{Digestible}\;\mathrm{IAA}\;\mathrm{reference}\;\mathrm{ratio}=\\ & \frac{\left(\mathrm{mg}\;\mathrm{digestible}\;\mathrm{IAA}\;/\;1\;g\;\mathrm{test}\;\mathrm{ingredient}\;\mathrm{crude}\;\mathrm{protein}\right)}{\left(\mathrm{mg}\;\mathrm{same}\;\mathrm{IAA}\;/\;1g\;\mathrm{crude}\;\;\mathrm{protein}\;\mathrm{requirement}\right)}. \end{array}$$ (1)

This equation produced a digestible IAA reference ratio for each IAA for a given reference IAA pattern; however, the DIAAS-like values reported herein for each ingredient were determined using the following equation wherein the lowest calculated digestible IAA reference ratio, which corresponds to the first or most LAA in the protein, is expressed as a percentage:


$$\begin{array}{ll} \mathrm{DIAAS}-\mathrm{like}\;\;\;\%\;= & \;100\times \mathrm{lowest}\;\mathrm{value}\\ & \;[\mathrm{digestibible}\;\mathrm{IAA}\;\mathrm{reference}\;\mathrm{ratio}]. \end{array}$$ (2)

Moreover, in accordance with the FAO, certain DIAAS-like thresholds were used to distinguish between ingredients that could be considered as an “excellent” quality protein source (DIAAS of ≥100), a “good” quality protein source (DIAAS of between 75 and 99), or ingredients that cannot carry a PQ claim at all (DIAAS of <75; FAO, 2013).

### Statistical analysis

All statistical analyses were performed using SAS (v. 9.4; SAS Institute Inc., Cary, NC, USA). For CP content, data were analyzed using PROC GLIMMIX of SAS with study (literature reference) of the nutritional data treated as a random effect, while “collective” AAFCO classification, “broad” AAFCO classification, regulatory body, and regulatory life stage as fixed effects. DIAAS-like data were analyzed using PROC GLIMMIX of SAS. The study (literature reference) responsible for the nutritional data was treated as a random effect, while collective AAFCO classification, broad AAFCO classification, “specific” AAFCO classification, regulatory body, regulatory life stage, and regulatory body*regulatory life stage were treated as fixed effects. For each of the aforementioned GLIMMIX procedures, when fixed effects were significant, means were separated using a Tukey adjustment. Statistical significance was declared at *P* ≤ 0.05.

## Results

### All ingredients

DIAAS-like values for all ingredients are presented in Table 4 (animal ingredients) and Table 5 (plant ingredients) along with the LAA associated with each DIAAS-like value (Tables 6 and 7, respectively).

**Table 4.** Digestible indispensable amino acid score (DIAAS)-like values and associated limiting amino acids (LAA) for animal ingredients (30 ingredients, 75 unique inputs) as determined using NRC, AAFCO, or FEDIAF amino acid requirements or recommendations at each discrete life stage as a reference pattern

Ingredient				NRC						AAFCO				FEDIAF					
Variety	Sort	Type	Ref^1^	AM^2^		EG		LG		AM		GR		AM		EG		LG	
Bovine	Beef	Lung meal	I	39	SAA^3^	82	SAA	85	SAA	70	SAA	67	THR	61	SAA	91	SAA	92	TRP
Bovine	Beef	Blood meal	G	9	ILE	12	ILE	12	ILE	16	ILE	11	ILE	14	ILE	13	ILE	14	ILE
Bovine	Beef	Loin	R	49	SAA	102	**TRP**	102	**TRP**	89	SAA	81	THR	76	SAA	111	**TRP**	97	TRP
Bovine	Beef	Ground	U	54	SAA	93	TRP	93	TRP	97	SAA	88	THR	83	SAA	101	**TRP**	88	TRP
Bovine	Milk	Casein	H	48	SAA	95	ARG	88	ARG	87	SAA	75	ARG	75	SAA	102	**ARG**	90	ARG
Bovine	Milk	Casein	G	40	SAA	84	SAA	86	ARG	72	SAA	73	ARG	62	SAA	93	SAA	88	ARG
Bovine	Milk	Casein	P	51	SAA	102	**ARG**	94	ARG	93	SAA	80	ARG	80	SAA	109	**ARG**	96	ARG
Bovine	Milk	Whole	P	51	SAA	98	ARG	90	ARG	92	SAA	77	ARG	79	SAA	104	**ARG**	92	ARG
Bovine	Milk	Whole	K	44	SAA	88	ARG	81	ARG	79	SAA	69	ARG	68	SAA	94	ARG	83	ARG
Bovine	Milk	Skimmed	P	51	SAA	97	ARG	90	ARG	92	SAA	76	ARG	79	SAA	104	**ARG**	92	ARG
Bovine	Milk	Skimmed	H	44	SAA	87	ARG	80	ARG	78	SAA	68	ARG	68	SAA	92	ARG	82	ARG
Bovine	Milk	Skimmed	O	44	SAA	92	SAA	89	ARG	79	SAA	76	ARG	68	SAA	102	**SAA**	91	ARG
Bovine	Milk	Whey protein concentrate	O	68	MET	87	ARG	81	ARG	108	**ARG**	69	ARG	100	**MET**	93	ARG	82	ARG
Bovine	Milk	Whey protein concentrate	H	63	SAA	70	ARG	65	ARG	87	ARG	55	ARG	86	ARG	75	ARG	66	ARG
Bovine	Milk	Whey protein concentrate	X	59	MET	68	ARG	63	ARG	84	ARG	54	ARG	84	ARG	73	ARG	65	ARG
Bovine	Milk	Whey	P	40	MET	62	ARG	57	ARG	72	MET	49	ARG	60	MET	66	ARG	58	ARG
Bovine	Milk	Whey	H	43	MET	64	ARG	59	ARG	76	MET	50	ARG	64	MET	68	ARG	60	ARG
Bovine	Milk	Whey	K	43	MET	53	ARG	49	ARG	66	ARG	42	ARG	64	MET	57	ARG	51	ARG
Marine	N/A	Meal	H	46	SAA	75	TRP	75	TRP	83	SAA	71	THR	72	SAA	82	TRP	72	TRP
Marine	N/A	Meal	G	45	SAA	87	TRP	87	TRP	81	SAA	69	THR	70	SAA	95	TRP	83	TRP
Marine	N/A	Meal	K	50	SAA	89	TRP	89	TRP	90	SAA	82	THR	78	SAA	97	TRP	85	TRP
Marine	N/A	Meal	P	49	SAA	94	TRP	94	TRP	88	SAA	80	THR	76	SAA	103	TRP	90	TRP
Marine	Pollock	Meat	R	55	SAA	92	TRP	92	TRP	99	SAA	80	THR	86	SAA	100	**TRP**	88	TRP
Marine	Salmon	Meat	R	50	SAA	101	**THR**	102	**TRP**	91	SAA	79	THR	78	SAA	111	**TRP**	97	TRP
Poultry	Chicken	Meat	M	37	SAA	76	SAA	79	SAA	66	SAA	70	THR	57	SAA	85	SAA	90	SAA
Poultry	Chicken	Meat	M	46	SAA	96	SAA	99	SAA	83	SAA	78	THR	71	SAA	107	SAA	99	TRP
Poultry	Chicken	Meat	M	38	SAA	79	SAA	82	SAA	68	SAA	68	THR	59	SAA	88	SAA	90	TRP
Poultry	Chicken	Meal	M	30	SAA	64	SAA	66	SAA	55	SAA	56	THR	47	SAA	71	SAA	67	TRP
Poultry	Chicken	Meal	V	45	SAA	94	TRP	94	TRP	81	SAA	75	THR	70	SAA	102	**TRP**	89	TRP
Poultry	Chicken	Meal	G	31	SAA	60	THR	61	THR	57	SAA	47	THR	49	SAA	67	TRP	58	TRP
Poultry	Chicken	Neck meal	I	43	SAA	77	THR	78	THR	77	SAA	60	THR	66	SAA	87	THR	87	THR
Poultry	Chicken	By-product meal	V	32	SAA	68	SAA	70	SAA	58	SAA	56	THR	50	SAA	75	SAA	79	TRP
Poultry	Chicken	Breast	R	47	SAA	98	THR	99	THR	85	SAA	77	THR	73	SAA	110	**THR**	103	**TRP**
Poultry	Egg	Whole	T	41	SAA	83	THR	84	THR	74	MET	65	THR	62	MET	93	THR	83	TRP
Poultry	Egg	Whole	S	50	SAA	104	**SAA**	106	**THR**	89	SAA	82	THR	77	SAA	115	**SAA**	118	**THR**
Poultry	N/A	By-product meal	I	40	SAA	83	TRP	83	TRP	72	SAA	69	THR	62	SAA	90	TRP	79	TRP
Poultry	N/A	By-product meal	I	36	SAA	74	SAA	77	SAA	64	SAA	60	THR	55	SAA	83	SAA	87	THR
Poultry	N/A	By-product meal	V	30	SAA	53	TRP	53	TRP	54	SAA	53	THR	47	SAA	57	TRP	50	TRP
Poultry	N/A	By-product meal	G	51	TRP	70	TRP	70	TRP	78	TRP	55	THR	73	TRP	76	TRP	66	TRP
Poultry	N/A	Meal	V	25	SAA	37	THR	37	THR	44	SAA	29	THR	38	SAA	41	THR	40	TRP
Poultry	N/A	Meal	Y	23	SAA	48	SAA	50	SAA	42	SAA	47	THR	36	SAA	54	SAA	49	TRP
Poultry	N/A	Feather meal	P	12	MET	26	MET	27	MET	22	MET	26	MET	19	MET	29	MET	31	MET
Poultry	N/A	Feather meal	K	16	MET	34	MET	35	MET	29	MET	33	HIS	24	MET	38	MET	41	MET
Poultry	N/A	Feather meal	Y	16	MET	23	LYS	22	LYS	25	LYS	22	LYS	24	MET	25	LYS	25	LYS
Poultry	N/A	Feather meal	H	17	MET	33	HIS	35	LYS	29	MET	29	HIS	25	MET	36	HIS	40	LYS
Poultry	N/A	Feather meal	V	13	MET	28	MET	29	MET	24	MET	28	MET	20	MET	31	MET	34	MET
Poultry	N/A	Feather meal	V	16	MET	34	MET	35	MET	29	MET	34	MET	24	MET	37	MET	40	MET
Poultry	N/A	Feather meal	J	30	TRP	41	TRP	41	TRP	46	TRP	42	HIS	43	TRP	44	TRP	39	TRP
Poultry	N/A	Feather meal	G	14	MET	29	MET	31	MET	25	MET	29	HIS	21	MET	33	MET	35	MET
Poultry	N/A	Feather meal	G	17	MET	35	MET	36	MET	3	MET	35	MET	25	MET	39	MET	42	MET
Swine	Pork	Liver meal	I	56	SAA	105	**THR**	106	**THR**	101	**SAA**	82	THR	87	SAA	117	**THR**	119	**THR**
Swine	Pork	Lung meal	I	43	SAA	90	SAA	93	SAA	77	SAA	77	THR	67	SAA	99	SAA	100	**TRP**
Swine	Pork	Loin	R	40	SAA	84	SAA	87	SAA	73	SAA	70	THR	63	SAA	94	SAA	86	TRP
Ovine	Lamb	Meal	I	29	SAA	60	SAA	62	SAA	52	SAA	54	THR	45	SAA	67	SAA	70	SAA
Ovine	Lamb	Lung meal	I	36	SAA	67	TRP	67	TRP	64	SAA	70	THR	55	SAA	73	TRP	64	TRP
N/A	N/A	Meat and bone meal	V	31	SAA	64	SAA	66	SAA	55	SAA	58	THR	47	SAA	71	SAA	63	TRP
N/A	N/A	Meat and bone meal	V	20	SAA	41	SAA	43	SAA	36	SAA	40	THR	31	SAA	46	SAA	42	TRP
N/A	N/A	Meat and bone meal	V	26	SAA	55	SAA	57	SAA	47	SAA	52	THR	40	SAA	61	SAA	58	TRP
N/A	N/A	Meat and bone meal	Y	21	SAA	43	SAA	45	SAA	37	SAA	43	SAA	32	SAA	48	SAA	51	SAA
N/A	N/A	Meat and bone meal	H	26	SAA	38	TRP	38	TRP	42	TRP	42	TRP	40	TRP	41	TRP	36	TRP
N/A	N/A	Meat and bone meal	G	27	SAA	57	SAA	59	SAA	49	SAA	57	SAA	42	SAA	63	SAA	60	TRP
N/A	N/A	Meat and bone meal	K	28	SAA	48	TRP	48	TRP	50	SAA	54	TRP	43	SAA	52	TRP	46	TRP
N/A	N/A	Meat and bone meal	P	14	TRP	19	TRP	19	TRP	21	TRP	21	TRP	20	TRP	21	TRP	18	TRP
N/A	N/A	Bone meal	P	19	TRP	25	TRP	25	TRP	29	TRP	29	TRP	27	TRP	28	TRP	24	TRP
N/A	N/A	Meat meal	P	24	SAA	49	SAA	51	SAA	42	SAA	49	SAA	36	SAA	54	SAA	49	TRP
N/A	N/A	Meat meal	H	28	SAA	54	TRP	54	TRP	50	SAA	54	THR	43	SAA	59	TRP	52	TRP
N/A	N/A	Meat meal	W	27	SAA	57	SAA	58	THR	49	SAA	44	THR	42	SAA	63	SAA	64	THR
N/A	N/A	Meat meal	V	30	SAA	62	SAA	64	SAA	53	SAA	56	THR	46	SAA	69	SAA	61	TRP
N/A	N/A	Meat meal	V	22	SAA	46	SAA	48	SAA	40	SAA	40	THR	34	SAA	51	SAA	52	TRP
N/A	N/A	Blood meal	P	24	ILE	31	ILE	32	ILE	43	ILE	29	ILE	36	ILE	35	ILE	36	ILE
N/A	N/A	Blood meal	K	27	SAA	40	ILE	40	ILE	48	SAA	36	ILE	42	SAA	44	ILE	46	ILE
N/A	N/A	Blood meal	G	16	ILE	21	ILE	21	ILE	28	ILE	19	ILE	24	ILE	23	ILE	24	ILE
N/A	N/A	Blood meal	H	21	ILE	28	ILE	28	ILE	38	ILE	25	ILE	32	ILE	31	ILE	32	ILE
N/A	N/A	Blood meal	V	18	ILE	23	ILE	24	ILE	32	ILE	21	ILE	27	ILE	26	ILE	27	ILE
N/A	N/A	Blood meal	V	33	SAA	65	ILE	65	ILE	60	SAA	59	ILE	51	SAA	72	ILE	75	ILE

Reference of nutritional data (amino acid profile, crude protein content, amino acid digestibility) for each ingredient; I, Cramer et al., 2007; G, Rojas and Stein, 2012; R, Faber et al., 2010; U, Bailey et al., 2020; H, NRC, 2012; P, CVB Feed Table, 2016; K, Sauvant et al., 2004; O, Mathai et al., 2017; X, Gottlob et al., 2006; M, Oba et al., 2019; V, Kerr et al., 2019; T, Zhang et al., 2015; S, Woyengo et al., 2015; Y, Park et al., 2020; J, Leme et al., 2019; W, Kong and Adeola, 2014.

AM, adult maintenance (using 110 kcal/kg^0.75^ for FEDIAF); EG, early growth (NRC, 4 to 14 wk of age; FEDIAF, less than 14 wk of age); LG, late growth (NRC and FEDAF, greater than 14 wk of age), GR, growth and reproduction (AAFCO only).

SAA, sulfur amino acids (Met + Cys).

Bolded limiting amino acids are not necessarily limiting.

**Table 5.** Digestible indispensable amino acid scores (DIAAS)-like values and associated limiting amino acids (LAA) for plant ingredients (27 ingredients, 94 unique inputs) as determined using NRC, AAFCO, or FEDIAF amino acid requirements or recommendations at each discrete life stage as a reference pattern

Ingredient				NRC						AAFCO				FEDIAF					
Variety	Sort	Type	Ref^1^	AM^2^		EG		LG		AM		GR		AM		EG		LG	
Grain	Barley	Whole	H	44	MET	67	THR	66	LYS	75	LYS	52	THR	65	MET	74	LYS	75	LYS
Grain	Barley	Whole	B	45	MET	79	THR	79	TRP	80	MET	61	THR	67	MET	86	TRP	75	TRP
Grain	Barley	Whole	K	44	MET	71	THR	71	LYS	79	MET	56	THR	66	MET	80	THR	81	THR
Grain	Barley	Whole	C	35	MET	57	THR	57	THR	62	MET	44	THR	52	MET	63	THR	64	THR
Grain	Canola	Meal	H	33	PHE	51	PHE	51	PHE	59	PHE	40	PHE	49	PHE	56	PHE	59	PHE
Grain	Canola	Meal	D	47	MET	79	THR	80	THR	83	MET	62	THR	70	MET	88	THR	90	THR
Grain	Cottonseed	Meal	D	22	MET	35	THR	35	THR	39	MET	27	THR	33	MET	39	THR	39	THR
Grain	Cottonseed	Meal	H	29	MET	61	MET	60	LYS	52	MET	51	THR	43	MET	68	MET	69	LYS
Grain	Corn	Germ meal	H	17	TRP	24	TRP	24	TRP	27	TRP	27	TRP	25	TRP	26	TRP	23	TRP
Grain	Corn	Germ meal	E	33	SAA^3^	59	TRP	59	TRP	60	SAA	52	THR	52	SAA	64	TRP	56	TRP
Grain	Corn	Germ meal	K	37	TRP	42	LYS	41	LYS	47	LYS	41	LYS	53	TRP	46	LYS	47	LYS
Grain	Corn	Gluten meal	J	22	TRP	31	TRP	31	TRP	34	TRP	34	TRP	32	TRP	33	TRP	29	TRP
Grain	Corn	Gluten meal	E	42	LYS	38	LYS	37	LYS	42	LYS	37	LYS	67	TRP	42	LYS	42	LYS
Grain	Corn	Gluten meal	B	23	TRP	31	TRP	31	TRP	35	TRP	35	TRP	33	TRP	34	TRP	30	TRP
Grain	Corn	Gluten meal	K	29	TRP	40	TRP	39	LYS	45	TRP	39	LYS	42	TRP	43	TRP	38	TRP
Grain	Corn	Gluten meal	P	32	TRP	40	LYS	38	LYS	44	LYS	38	LYS	46	TRP	44	LYS	42	TRP
Grain	Corn	Whole	H	44	TRP	57	LYS	56	LYS	64	LYS	56	LYS	63	TRP	63	LYS	57	TRP
Grain	Corn	Whole	J	48	SAA	66	TRP	66	TRP	75	TRP	65	THR	70	TRP	72	TRP	63	TRP
Grain	Corn	Whole	E	38	TRP	53	TRP	53	TRP	59	TRP	57	LYS	56	TRP	57	TRP	50	TRP
Grain	Corn	Whole	C	36	TRP	49	TRP	49	TRP	55	TRP	46	THR	51	TRP	53	TRP	46	TRP
Grain	Corn	Whole	K	37	TRP	51	TRP	51	TRP	58	TRP	58	TRP	54	TRP	56	TRP	49	TRP
Grain	Corn	Whole	P	38	TRP	52	TRP	52	TRP	59	TRP	54	LYS	55	TRP	57	TRP	50	TRP
Grain	Corn	DDGS	E	39	LYS	35	LYS	34	LYS	39	LYS	34	LYS	62	LYS	39	LYS	39	LYS
Grain	Corn	DDGS	H	41	TRP	44	LYS	43	LYS	49	LYS	43	LYS	59	TRP	49	LYS	49	LYS
Grain	Flaxseed	Meal	H	40	PHE	55	AAA	56	AAA	73	PHE	49	PHE	61	PHE	61	AAA	64	AAA
Grain	Flaxseed	Meal	D	26	VAL	43	VAL	40	VAL	47	VAL	43	VAL	40	VAL	47	VAL	46	VAL
Grain	Flaxseed	Meal	P	49	SAA	78	LEU	75	LYS	86	LYS	62	THR	74	MET	86	LYS	86	LYS
Grain	Oat	Whole	H	45	MET	74	THR	75	THR	80	MET	58	THR	67	MET	83	THR	84	THR
Grain	Oat	Whole	K	47	MET	67	THR	68	THR	82	MET	52	THR	69	MET	75	THR	76	THR
Grain	Oat	Whole	P	42	MET	73	THR	74	THR	75	MET	57	THR	63	MET	82	THR	83	THR
Grain	Oat	Groats	C	44	TRP	60	TRP	60	TRP	68	TRP	58	THR	63	TRP	66	TRP	57	TRP
Grain	Oat	Groats	H	36	MET	65	LYS	63	LYS	63	MET	51	THR	53	MET	72	LYS	72	LYS
Grain	Oat	Groats	K	47	MET	77	THR	78	THR	82	MET	60	THR	69	MET	86	THR	87	THR
Grain	Peanut	Meal	H	28	MET	45	LEU	55	LEU	50	MET	45	LEU	42	MET	50	LEU	64	LEU
Grain	Peanut	Meal	J	37	MET	75	THR	76	THR	66	MET	58	THR	55	MET	83	THR	81	TRP
Grain	Rice	Bran	H	42	SAA	73	THR	74	THR	76	SAA	57	THR	65	SAA	81	THR	82	THR
Grain	Rice	Bran	B	44	TRP	60	TRP	60	TRP	68	TRP	54	THR	63	TRP	66	TRP	58	TRP
Grain	Rice	Whole	H	58	THR	68	THR	69	THR	92	THR	53	THR	86	THR	76	THR	77	THR
Grain	Rice	Whole	P	52	SAA	75	THR	75	THR	93	SAA	58	THR	79	MET	83	THR	80	TRP
Grain	Rice	Whole	P	60	MET	97	THR	98	THR	106	**MET**	75	THR	89	MET	108	**THR**	101	**TRP**
Grain	Rice	Whole	C	5	TRP	73	TRP	73	TRP	82	TRP	58	THR	77	TRP	79	TRP	69	TRP
Grain	Rye	Whole	H	25	PHE	39	PHE	40	PHE	45	PHE	31	PHE	38	PHE	44	PHE	45	PHE
Grain	Rye	Whole	C	34	MET	48	THR	49	THR	61	MET	38	THR	51	MET	54	THR	52	TRP
Grain	Rye	Whole	K	39	MET	69	THR	69	THR	69	MET	54	THR	57	MET	77	THR	70	TRP
Grain	Rye	Whole	P	40	MET	68	THR	69	THR	71	MET	53	THR	60	MET	76	THR	71	TRP
Grain	Sorghum	Whole	H	41	SAA	40	LYS	39	LYS	44	LYS	39	LYS	59	TRP	44	LYS	44	LYS
Grain	Sorghum	Whole	C	29	TRP	37	LYS	35	LYS	41	LYS	35	LYS	41	TRP	40	LYS	37	TRP
Grain	Sorghum	Whole	K	41	MET	44	LYS	43	LYS	49	LYS	43	LYS	61	MET	48	LYS	49	LYS
Grain	Sorghum	Whole	P	49	SAA	50	LYS	48	LYS	55	LYS	48	LYS	73	MET	55	LYS	55	LYS
Grain	Sunflower	Meal	D	41	SAA	68	LYS	66	LYS	74	SAA	58	THR	64	SAA	75	LYS	76	LYS
Grain	Sunflower	Meal	K	54	SAA	74	LYS	72	LYS	83	LYS	64	THR	84	SAA	82	LYS	83	LYS
Grain	Wheat	Bran	H	32	MET	65	LYS	63	LYS	57	MET	55	THR	48	MET	72	LYS	73	LYS
Grain	Wheat	Bran	J	37	MET	67	THR	68	THR	65	MET	53	THR	54	MET	75	THR	76	THR
Grain	Wheat	Bran	B	33	MET	53	TRP	53	TRP	59	MET	48	THR	49	MET	58	TRP	50	TRP
Grain	Wheat	Bran	K	36	MET	57	THR	58	THR	63	MET	45	THR	53	MET	64	THR	65	THR
Grain	Wheat	Middlings	H	40	MET	67	THR	68	THR	71	MET	53	THR	59	MET	75	THR	76	THR
Grain	Wheat	Middlings	K	39	MET	64	THR	64	THR	69	MET	50	THR	58	MET	71	THR	72	THR
Grain	Wheat	Middlings	P	42	MET	67	THR	67	THR	74	MET	52	THR	62	MET	74	THR	75	THR
Grain	Wheat	DDGS	H	29	LYS	26	LYS	26	LYS	29	LYS	26	LYS	47	LYS	29	LYS	29	LYS
Grain	Wheat	DDGS	K	37	LYS	33	LYS	32	LYS	37	LYS	32	LYS	59	LYS	37	LYS	37	LYS
Grain	Wheat	Whole	O	42	MET	74	THR	75	THR	74	MET	58	THR	62	MET	83	THR	75	TRP
Grain	Wheat	Whole	H	42	MET	57	LYS	55	LYS	63	LYS	50	THR	62	MET	63	LYS	63	LYS
Grain	Wheat	Whole	B	50	MET	69	TRP	69	TRP	77	TRP	57	THR	72	TRP	75	TRP	65	TRP
Grain	Wheat	Whole	C	42	THR	49	THR	50	THR	60	LYS	38	THR	62	THR	55	THR	56	THR
Grain	Wheat	Whole	P	45	MET	60	LYS	58	LYS	66	LYS	53	THR	66	MET	66	LYS	66	LYS
Legume	Soybean	Meal	H	38	SAA	80	SAA	82	SAA	68	MET	72	THR	57	MET	88	SAA	93	SAA
Legume	Soybean	Meal	W	44	TRP	60	TRP	60	TRP	68	TRP	68	TRP	63	TRP	66	TRP	58	TRP
Legume	Soybean	Meal	J	34	MET	71	MET	73	MET	6	MET	71	MET	50	MET	79	MET	85	MET
Legume	Soybean	Meal	B	41	MET	85	MET	88	MET	72	MET	81	THR	60	MET	94	MET	90	TRP
Legume	Soybean	Meal	K	40	MET	83	MET	86	MET	71	MET	75	THR	59	MET	93	MET	98	SAA
Legume	Soybean	Protein concentrate	H	36	SAA	75	SAA	77	SAA	64	SAA	72	THR	55	SAA	83	SAA	88	SAA
Legume	Soybean	Protein concentrate	D	26	VAL	42	VAL	39	VAL	46	VAL	41	VAL	39	VAL	46	VAL	45	VAL
Legume	Soybean	Protein concentrate	J	35	MET	73	MET	75	MET	62	MET	73	MET	52	MET	81	MET	87	MET
Legume	Lupin bean	Whole	B	19	MET	39	MET	40	MET	33	MET	39	MET	27	MET	43	MET	46	MET
Legume	Fava bean	Whole	H	16	MET	34	MET	35	MET	28	MET	34	MET	24	MET	37	MET	40	MET
Legume	Fava bean	Whole	K	18	MET	38	MET	39	MET	32	MET	38	MET	27	MET	42	MET	45	MET
Legume	Fava bean	Whole	K	18	MET	37	MET	38	MET	31	MET	37	MET	26	MET	41	MET	44	MET
Legume	Fava bean	Whole	P	16	MET	34	MET	35	MET	29	MET	34	MET	24	MET	38	MET	40	MET
Legume	Fava bean	Whole	P	21	MET	44	MET	46	MET	38	MET	44	MET	31	MET	49	MET	53	MET
Legume	Pea	Whole	K	24	MET	49	MET	51	MET	42	MET	50	MET	35	MET	55	MET	59	MET
Legume	Pea	Whole	P	22	MET	47	MET	48	MET	40	MET	47	MET	33	MET	52	MET	56	MET
Legume	Pea	Whole	H	23	MET	47	MET	49	MET	40	MET	47	MET	34	MET	53	MET	57	MET
Legume	Pea	Whole	F	18	MET	37	MET	39	MET	32	MET	37	MET	26	MET	41	MET	44	MET
Legume	Pea	Whole	F	18	MET	37	MET	39	MET	32	MET	37	MET	27	MET	42	MET	45	MET
Legume	Pea	Whole	F	24	MET	50	MET	52	MET	43	MET	50	MET	36	MET	56	MET	60	MET
Legume	Pea	Whole	F	22	MET	46	MET	48	MET	39	MET	46	MET	33	MET	51	MET	53	TRP
Tuber	Potato	Protein concentrate	H	58	MET	109	**TRP**	109	**TRP**	102	**MET**	99	HIS	86	MET	119	**TRP**	104	**TRP**
Tuber	Potato	Protein concentrate	D	33	VAL	53	VAL	50	VAL	58	VAL	53	VAL	49	VAL	59	VAL	57	VAL
Tuber	Potato	Protein concentrate	K	47	SAA	92	TRP	92	TRP	85	SAA	99	SAA	73	SAA	99	TRP	88	TRP
Tuber	Potato	Protein concentrate	P	50	SAA	104	**SAA**	108	**SAA**	90	SAA	98	HIS	77	SAA	1156	**SAA**	107	**TRP**
Tuber	Potato	Protein concentrate	P	50	SAA	104	**SAA**	108	**SAA**	90	SAA	98	HIS	77	SAA	116	**SAA**	106	**TRP**
Yeast	Yeast	Brewers	P	32	SAA	66	SAA	69	SAA	57	SAA	66	SAA	49	SAA	73	SAA	78	SAA
Yeast	Yeast	Brewers	K	32	MET	59	TRP	59	TRP	57	MET	62	THR	47	MET	64	TRP	56	TRP
Yeast	Yeast	Brewers	H	25	AAA	32	AAA	32	AAA	45	AAA	31	PHE	39	AAA	35	AAA	37	AAA

Reference of nutritional data (amino acid profile, crude protein content, amino acid digestibility) for each ingredient; H, NRC, 2012; B, Lee et al., 2020; K, Sauvant et al., 2004; C, Cervantes-Pahm et al., 2014; D, Cotten et al., 2016; E, Almeida et al., 2011; J, Leme et al., 2019; P, CVB Feed Table, 2016; O, Mathai et al., 2017; W, Kong and Adeola, 2014; F, Grosjean et al., 2000.

AM, adult maintenance (using 110 kcal/kg^0.75^ for FEDIAF); EG, early growth (NRC, 4 to 14 wk of age; FEDIAF, less than 14 wk of age); LG, late growth (NRC and FEDAF, greater than 14 wk of age), GR, growth and reproduction (AAFCO only).

AAA, aromatic amino acids (Phe + Tyr); SAA, sulfur amino acids (Met + Cys).

Bolded limiting amino acids are not necessarily limiting.

**Table 6.** Frequency of limiting amino acids for animal ingredients (*n* = 75) as determined using NRC, AAFCO, or FEDIAF^1^ amino acid requirements or recommendations at each discrete life stage as a reference pattern

AA^2^	NRC			AAFCO		FEDIAF			Total
	AM^3^	EG	LG	AM	GR	AM	EG	LG	
Arg	0	12	14	4	14	2	12	14	72
His	0	1	0	0	4	0	1	0	6
Ile	5	7	7	5	7	5	7	7	50
Leu	0	0	0	0	0	0	0	0	0
Lys	0	1	2	1	1	0	1	2	8
Phe	0	0	0	0	0	0	0	0	0
AAA	0	0	0	0	0	0	0	0	0
Met	13	6	6	10	4	13	6	6	64
SAA	53	23	19	50	3	50	23	3	224
Thr	0	7	8	0	38	0	5	5	63
Trp	4	18	19	5	4	5	20	38	113
Val	0	0	0	0	0	0	0	0	0

NRC, National Research Council Nutrient Requirements of Dogs and Cats (2006); AAFCO, Association of American Feed Control Officials (2016); FEDIAF, European Pet Food Industry Federation Nutritional Guidelines for Complete and Complementary Pet Foods for Cats and Dogs (2018).

AA, amino acid; AAA, aromatic amino acids (Phe + Tyr); SAA, sulfur amino acids (Met + Cys).

AM, adult maintenance (using 110 kcal/kg^0.75^ for FEDIAF); EG, early growth (NRC, 4 to 14 wk of age; FEDIAF, less than 14 wk of age); LG, late growth (NRC and FEDAF, greater than 14 wk of age), GR, growth and reproduction (AAFCO only).

**Table 7.** Frequency of limiting amino acids for plant ingredients (*n* = 94) as determined using NRC, AAFCO, or FEDIAF^1^ amino acid requirements or recommendations at each discrete life stage as a reference pattern

AA^2^	NRC			AAFCO		FEDIAF			Total
	AM^3^	EG	LG	AM	GR	AM	EG	LG	
Arg	0	0	0	0	0	0	0	0	0
His	0	0	0	0	3	0	0	0	3
Ile	0	0	0	0	0	0	0	0	0
Leu	0	2	1	0	1	0	1	1	6
Lys	4	18	23	18	15	3	20	18	119
Phe	3	2	2	3	4	3	2	2	21
AAA	1	2	2	1	0	1	2	2	11
Met	49	18	17	45	15	52	18	14	228
SAA	15	5	5	9	2	9	5	4	54
Thr	2	25	22	1	46	2	23	16	137
Trp	17	19	19	14	5	21	20	34	149
Val	3	3	3	3	3	3	3	3	24

NRC, National Research Council Nutrient Requirements of Dogs and Cats (2006); AAFCO, Association of American Feed Control Officials (2016); FEDIAF, European Pet Food Industry Federation Nutritional Guidelines for Complete and Complementary Pet Foods for Cats and Dogs (2018).

AA, amino acid; AAA, aromatic amino acids (Phe + Tyr); SAA, sulfur amino acids (Met + Cys).

AM, adult maintenance (using 110 kcal/kg^0.75^ for FEDIAF); EG, early growth (NRC, 4 to 14 wk of age; FEDIAF, less than 14 wk of age); LG, late growth (NRC and FEDAF, greater than 14 wk of age), GR, growth and reproduction (AAFCO only).

### All ingredients—NRC reference patterns

For animal ingredients, when using NRC estimated minimal requirements for AM, EG, and LG as reference patterns, there were 0, 25, and 27 respective cases wherein DIAAS-like values of between 75 and 99 (“good” quality protein source) were achieved, and there were 0, 5, and 4 respective cases wherein DIAAS-like values of ≥100 (“excellent” quality protein source) were achieved (Table 4). The most prevalent LAA when using NRC requirements for AM, EG, and LG as reference IAA patterns were SAA (53 instances), SAA (23 instances), and Trp (19 instances), respectively (Table 6).

For plant ingredients, when using NRC requirements for AM, EG, and LG as reference IAA patterns, there were 0, 8, and 15 respective cases wherein DIAAS-like values of between 75 and 99 were achieved, and there were 0, 3, and 3 respective cases wherein DIAAS-like values of ≥100 were achieved (Table 5). The most prevalent LAA when using NRC requirements for AM, EG, and LG as reference IAA patterns were Met (49 instances), Thr (25 instances), and Lys (23 instances), respectively (Table 7).

### All ingredients—AAFCO reference patterns

For animal ingredients, when using AAFCO recommendations for AM and GR as reference IAA patterns, there were 24 and 17 respective cases wherein DIAAS-like values of 75 to 99 were achieved, and there were 2 and 0 respective cases wherein DIAAS-like values of ≥100 were achieved (Table 4). The most prevalent LAA when using AAFCO (2016) requirements for AM and GR as reference IAA patterns were SAA (50 instances) and Thr (38 instances), respectively (Table 6).

For plant ingredients, when using AAFCO recommendations for AM and GR as reference IAA patterns, there were 16 and 5 respective cases wherein DIAAS-like values of 75 to 99 were achieved, and there were 2 and 0 respective cases wherein DIAAS-like values of ≥100 were achieved (Table 5). The most prevalent LAA when using AAFCO recommendations for AM and GR as reference IAA patterns were Met (45 instances) and Thr (46 instances), respectively (Table 7).

### All ingredients—FEDIAF reference patterns

For animal ingredients, when using FEDIAF recommendations for AM, EG, and LG as reference IAA patterns, there were 13, 18, and 27 respective cases wherein DIAAS-like values of between 75 and 99 were achieved, and there were 1, 14, and 4 respective cases wherein DIAAS-like values of ≥100 were achieved (Table 4). The most prevalent LAA when using FEDIAF recommendations for AM, EG, and LG as reference IAA patterns were SAA (50 instances), SAA (23 instances), and Trp (38 instances), respectively (Table 6).

For plant ingredients, when using FEDIAF recommendations for AM, EG, and LG as reference IAA patterns, there were 8, 24, and 24 respective cases wherein DIAAS-like values of between 75 and 99 were achieved, and there were 0, 4, and 4 respective cases wherein DIAAS-like values of ≥100 were achieved (Table 5). The most prevalent LAA when using FEDIAF recommendations for AM, EG, and LG as reference IAA patterns were Met (52 instances), Thr (23 instances), and Trp (34 instances), respectively (Table 7).

### All ingredients—reference pattern comparison

Table 8 summarizes the DIAAS-like values for all ingredients combined as determined using NRC, AAFCO, and FEDIAF IAA requirements or recommendations at each life stage. When considering all ingredients together, regardless of plant or animal origin, the greatest DIAAS-like value was achieved using the FEDIAF EG reference pattern, while the lowest DIAAS-like value was achieved using NRC AM (*P* ≤ 0.05).

**Table 8.** Least squares means (± SEM) for digestible indispensable amino acid score (DIAAS)-like values for all ingredients as determined using NRC, AAFCO, or FEDIAF^1^ amino acid requirements or recommendations at each discrete life stage as a reference pattern

NRC			AAFCO		FEDIAF			SEM	*P*-value
AM^2^	EG	LG	AM	GR	AM	EG	LG		
38.93^d^	63.65^b^	63.82^ab^	63.50^b^	56.58^c^	56.95^c^	69.72^a^	67.05^ab^	3.51	<0.01

NRC, National Research Council Nutrient Requirements of Dogs and Cats (2006); AAFCO, Association of American Feed Control Officials (2016); FEDIAF, European Pet Food Industry Federation Nutritional Guidelines for Complete and Complementary Pet Foods for Cats and Dogs (2018).

AM, adult maintenance; EG, early growth (NRC, 4 to 14 wk of age; FEDIAF, less than 14 wk of age); LG, late growth (NRC and FEDAF, greater than 14 wk of age), GR, growth and reproduction (AAFCO only).

Values in a row with different superscript are significantly different (*P* ≤ 0.05).

### Animal ingredients—specific AAFCO classification

Table 9 summarizes the DIAAS-like values for the animal ingredients determined using NRC, AAFCO, and FEDIAF IAA requirements or recommendations at each life stage for the specific AAFCO ingredient category. No differences were observed for DIAAS-like values between any life stage (*P* > 0.05) for “blood meal”, “fish meal”, or “poultry meal”. For “animal by-product meal”, the greatest DIAAS-like values were achieved using NRC and FEDIAF EG and LG reference patterns, while the lowest DIAAS-like values were achieved using NRC AM (*P* ≤ 0.05). The FEDIAF EG reference pattern generated the greatest DIAAS-like values for “casein”, “dried milk”, “dried skimmed milk”, “meat”, “meat and bone meal”, and “meat meal”, while the lowest DIAAS-like values were achieved using NRC AM (*P* ≤ 0.05). For “egg product” and “poultry by-product meal”, the greatest DIAAS-like values were achieved using FEDIAF EG and LG reference patterns, while the lowest DIAAS-like values were achieved using NRC AM (*P* ≤ 0.05). The AAFCO and FEDIAF AM reference patterns produced the greatest DIAAS-like values for “dried whey concentrate”, while the lowest DIAAS-like values were achieved using AAFCO GR (*P* ≤ 0.05). For “dried whey”, the greatest DIAAS-like values were achieved using the AAFCO AM reference pattern, while the lowest DIAAS-like value was achieved using AAFCO GR and NRC AM (*P* ≤ 0.05). Last, the FEDIAF LG reference pattern resulted in the greatest DIAAS-like values for “hydrolyzed poultry feathers”, while the lowest DIAAS-like value was achieved using NRC AM (*P* ≤ 0.05).

**Table 9.** Least squares means (± SEM) for digestible indispensable amino acid score (DIAAS)-like values as determined using NRC, AAFCO, or FEDIAF^1^ amino acid requirements or recommendations at each discrete life stage as a reference pattern for animal ingredients categorized based on specific AAFCO Official Common and Usual Names and Definitions of Feed Ingredients

Specific AAFCO classification	NRC			AAFCO			FEDIAF		SEM	*P*-value
	AM^2^	EG	LG	AM	GR	AM	EG	LG		
Animal by-product meal	22.88^b^	58.29^a^	59.79^a^	52.68^ab^	49.29^ab^	43.65^ab^	66.12^a^	64.17^a^	27.00	<0.01
Blood meal	21.87	31.96	32.31	38.52	29.32	32.72	35.44	36.83	6.25	0.26
Casein	46.56^f^	93.81^ab^	89.51^bc^	83.82^cd^	76.24^de^	72.23^e^	101.33^a^	91.58^bc^	4.12	<0.01
Dried milk	47.28^e^	92.79^b^	85.79^c^	85.11^c^	73.08^d^	73.34^d^	99.02^a^	87.78^bc^	4.82	<0.01
Dried skimmed milk	46.25^e^	91.86^ab^	86.35^bc^	83.24^c^	73.56^d^	71.74^d^	99.38^a^	88.36^bc^	3.32	<0.01
Dried whey concentrate	63.09^de^	75.12^bc^	69.45^cd^	92.80^a^	59.16^e^	89.86^a^	80.16^b^	71.06^c^	5.62	<0.01
Dried whey	42.13^d^	59.56^bc^	55.07^c^	71.30^a^	46.91^d^	62.50^bc^	63.56^ab^	56.34^bc^	2.66	<0.01
Egg product	45.42^d^	93.39^ab^	94.80^ab^	81.67^abc^	73.25^bc^	69.46^c^	103.91^a^	100.15^a^	10.40	<0.01
Fish meal	44.30	79.08	79.34	77.22	69.53	66.89	85.99	76.18	11.27	0.13
Hydrolyzed poultry feathers	16.89^e^	31.50^abc^	32.37^abc^	28.79^cd^	30.95^bc^	24.87^d^	34.83^ab^	36.36^a^	2.41	<0.01
Meat	40.46^c^	79.43^ab^	80.67^ab^	73.68^ab^	68.32^ab^	64.21^b^	87.60^a^	80.75^ab^	11.68	<0.01
Meat and bone meal	22.09^c^	43.67^ab^	44.87^ab^	40.29^ab^	44.09^ab^	35.04^b^	48.41^a^	44.83^ab^	5.28	<0.01
Meat meal	26.63^e^	54.82^abc^	56.29^abc^	47.82^cd^	49.65^bcd^	41.23^d^	60.68^a^	58.19^ab^	2.60	<0.01
Poultry by-product meal	38.06^c^	70.26^ab^	71.28^ab^	66.78^ab^	58.44^b^	58.41^b^	77.34^a^	74.20^a^	5.39	<0.01
Poultry meal	30.55	60.16	61.20	55.28	50.27	47.59	66.36	60.35	8.06	0.08

NRC, National Research Council Nutrient Requirements of Dogs and Cats (2006); AAFCO, Association of American Feed Control Officials (2016); FEDIAF, European Pet Food Industry Federation Nutritional Guidelines for Complete and Complementary Pet Foods for Cats and Dogs (2018).

AM, adult maintenance; EG, early growth (NRC, 4 to 14 wk of age; FEDIAF, less than 14 wk of age); LG, late growth (NRC and FEDAF, greater than 14 wk of age), GR, growth and reproduction (AAFCO only).

Values in a row with different superscript are significantly different (*P* ≤ 0.05).

### Plant ingredients—specific AAFCO classification

Table 10 summarizes the DIAAS-like values for the plant ingredients determined using NRC, AAFCO, and FEDIAF IAA requirements or recommendations at each life stage for the specific AAFCO ingredient category. For “barley grain” and “potato protein”, the greatest DIAAS-like values were achieved using FEDIAF EG reference patterns, while the lowest DIAAS-like values were achieved using NRC AM (*P* ≤ 0.05). For “brewers dried yeast”, the greatest DIAAS-like values were achieved using NRC EG and LG, AAFCO AM and GR, and FEDIAF EG and LG reference patterns, while the lowest DIAAS-like value was achieved using NRC AM (*P* ≤ 0.05). The greatest DIAAS-like values for “canola meal”, “linseed meal”, “oat grain”, “rice bran”, and “sunflower meal”, were achieved using AAFCO AM and FEDIAF EG and LG reference patterns, while the lowest DIAAS-like values were achieved using NRC AM (*P* ≤ 0.05). The greatest DIAAS-like values for “corn germ meal”, “corn gluten meal”, and “rice grain” were generated using AAFCO AM and FEDIAF AM and EG reference patterns, while the lowest DIAAS-like values were achieved using NRC AM (*P* ≤ 0.05). For “corn grain”, the greatest DIAAS-like values were produced using the AAFCO AM reference pattern, while the lowest DIAAS-like values were achieved using NRC AM (*P* ≤ 0.05). For “cottonseed meal”, “oat groats”, “soy protein concentrate”, “soybean meal”, and “wheat bran”, the greatest DIAAS-like values were achieved using FEDIAF EG and LG reference patterns, while the lowest DIAAS-like values were generated using NRC AM (*P* ≤ 0.05). For “DDGS”, the greatest DIAAS-like value was achieved using FEDIAF AM requirements, while all other reference patterns resulted in lesser DIAAS-like values (*P* ≤ 0.05). The greatest DIAAS-like values were generated for “dried beans”, “dried peas”, and “wheat middlings” using the FEDIAF LG reference pattern, while the lowest DIAAS-like values were achieved using NRC AM (*P* ≤ 0.05). For “rye grain”, the greatest DIAAS-like values were achieved using FEDIAF EG and AAFCO AM reference patterns, while the lowest DIAAS-like value was produced using NRC AM (*P* ≤ 0.05). Last, for “sorghum grain”, the greatest DIAAS-like value was generated using the FEDIAF AM reference pattern, while the lowest DIAAS-like value was achieved using NRC AM (*P* ≤ 0.05).

**Table 10.** Least squares means (± SEM) for digestible indispensable amino acid score (DIAAS)-like values as determined using NRC, AAFCO, or FEDIAF^1^ amino acid requirements or recommendations at each discrete life stage as a reference pattern for plant ingredients categorized based on specific AAFCO Official Common and Usual Names and Definitions of Feed Ingredients

Specific AAFCO classification	NRC			AAFCO		FEDIAF			SEM	*P*-value
	AM^2^	EG	LG	AM	GR	AM	EG	LG		
Barley grain	42.23^e^	68.36^bc^	68.13^bc^	74.04^ab^	53.41^d^	62.65^c^	75.71^a^	73.61^ab^	3.92	<0.01
Brewers dried yeast	29.48^b^	52.11^a^	53.04^a^	52.73^a^	52.87^a^	44.62^ab^	57.43^a^	56.66^a^	9.05	<0.01
Canola meal	39.76^c^	64.95^ab^	65.68^ab^	70.93^a^	50.79^bc^	59.26^ab^	72.31^a^	73.98^a^	13.01	<0.01
Corn germ meal	29.13^b^	41.51^ab^	41.12^ab^	44.48^a^	39.78^ab^	43.25^a^	45.37^a^	41.75^ab^	9.29	0.03
Corn gluten meal	29.59^b^	35.82^ab^	35.30^ab^	40.08^a^	36.84^ab^	43.90^a^	39.21^a^	36.13^ab^	3.19	0.03
Corn grain	40.04^f^	54.70^de^	54.44^de^	61.41^a^	55.69^cd^	57.95^bc^	59.63^ab^	52.56^e^	2.60	<0.01
Cottonseed meal	25.52^b^	47.65^ab^	47.51^ab^	45.34^ab^	39.00^ab^	37.86^ab^	53.01^a^	53.82^a^	11.10	0.04
DDGS^3^	36.37^b^	34.65^b^	33.68^b^	38.50^b^	33.68^b^	56.56^a^	38.28^b^	38.49^b^	3.66	<0.01
Dried bean	17.66^e^	37.18^bc^	38.56^ab^	31.51^cd^	37.18^bc^	26.33^d^	41.34^ab^	44.54^a^	1.68	<0.01
Dried pea	22.75^e^	46.17^bc^	47.83^ab^	39.36^cd^	46.17^bc^	33.15^d^	51.16^ab^	54.67^a^	2.15	<0.01
Linseed meal	38.53^c^	58.52^ab^	57.08^ab^	68.71^a^	51.09^bc^	58.10^ab^	64.77^a^	65.30^a^	9.85	<0.01
Oat grain	44.66^d^	71.57^ab^	72.36^ab^	79.17^a^	55.91^c^	66.26^b^	79.76^a^	80.76^a^	2.16	<0.01
Oat groats	41.92^c^	67.43^ab^	67.12^ab^	71.01^ab^	56.40^bc^	61.61^ab^	74.39^a^	72.19^a^	5.44	<0.01
Peanut meal	32.52^b^	59.71^a^	65.32^a^	57.64^a^	51.49^ab^	48.25^ab^	66.42^a^	72.64^a^	10.34	<0.01
Potato protein	45.08^d^	90.10^ab^	90.94^ab^	82.64^bc^	86.86^ab^	70.13^c^	99.32^a^	89.94^ab^	10.93	<0.01
Rice bran	42.98^b^	66.51^ab^	66.91^ab^	71.80^a^	55.34^ab^	64.31^ab^	73.29^a^	69.74^a^	6.24	0.02
Rice grain	53.93^c^	76.42^ab^	77.08^ab^	91.62^a^	59.73^bc^	80.99^a^	84.85^a^	80.13^ab^	5.77	<0.01
Rye grain	34.59^d^	55.98^ab^	56.60^ab^	61.49^a^	43.75^c^	51.42^bc^	62.36^a^	59.67^ab^	6.36	<0.01
Sorghum grain	39.65^c^	42.42^bc^	41.24^bc^	47.13^b^	41.24^bc^	58.63^a^	46.86^b^	39.65^bc^	3.80	<0.01
Soy protein concentrate	32.04^c^	62.96^ab^	63.85^ab^	57.27^ab^	61.99^ab^	48.52^bc^	69.84^a^	73.03^a^	9.91	<0.01
Soybean meal	39.24^d^	75.71^ab^	78.07^ab^	67.60^bc^	73.16^ab^	57.89^c^	83.83^a^	84.73^a^	4.23	<0.01
Sunflower meal	47.60^c^	71.34^ab^	69.36^ab^	78.27^a^	61.23^bc^	73.84^ab^	78.82^a^	79.27^a^	5.22	<0.01
Wheat bran	34.42^d^	60.67^abc^	60.56^abc^	61.01^ab^	50.03^c^	51.06^bc^	67.10^a^	65.85^a^	3.21	<0.01
Wheat grain	43.85^b^	61.68^a^	61.31^a^	68.06^a^	51.17^b^	64.75^a^	68.14^a^	65.03^a^	3.58	<0.01
Wheat middlings	40.06^f^	65.81^c^	66.55^c^	71.02^b^	51.42^e^	59.44^d^	73.35^ab^	74.27^a^	1.20	<0.01

NRC, National Research Council Nutrient Requirements of Dogs and Cats (2006); AAFCO, Association of American Feed Control Officials (2016); FEDIAF, European Pet Food Industry Federation Nutritional Guidelines for Complete and Complementary Pet Foods for Cats and Dogs (2018).

AM, adult maintenance; EG, early growth (NRC, 4 to 14 wk of age; FEDIAF, less than 14 wk of age); LG, late growth (NRC and FEDAF, greater than 14 wk of age), GR, growth and reproduction (AAFCO only).

DDGS, distillers grain with solubles.

Values in a row with different superscript are significantly different (*P* ≤ 0.05).

### Broad AAFCO ingredient classification

Supplementary Table S1 summarizes the DIAAS-like values for all broad AAFCO ingredient categories of animal ingredients determined using NRC, AAFCO, and FEDIAF IAA requirements or recommendations at each life stage. Regardless of the organization (NRC, AAFCO, FEDIAF) or life stage (AM, EG, LG, GR) used as a reference pattern, “marine products” and “milk products” produced greater DIAAS-like values than “animal products” (*P* ≤ 0.05). In addition, “animal products” and “marine products” had greater CP (% DM basis) than “milk products” (*P* ≤ 0.05).

Supplementary Table S2 summarizes the DIAAS-like values for all broad AAFCO ingredient categories of plant ingredients determined using NRC, AAFCO, and FEDIAF IAA requirements or recommendations at each life stage. Briefly, differences were observed for DIAAS-like values and CP content for each broad AAFCO category, regardless of the reference pattern utilized. Refer to Supplementary Material S2 for a more detailed overview of the collective AAFCO ingredient category results.

### Collective AAFCO ingredient classification

Supplementary Table S3 summarizes the DIAAS-like values for each collective AAFCO ingredient category of plant ingredients (“plant protein products”, “processed grain by-products”, “grain products”). In addition, this table summarizes the comparisons between the collective AAFCO ingredient categories of “animal protein products” and “plant protein products”. In brief, no differences were observed between the mean DIAAS-like values of “animal protein products” and “plant protein products”, regardless of the reference pattern (*P* > 0.05). Refer to Supplementary Material S3 for a more detailed breakdown of the collective AAFCO ingredient category results.

## Discussion

The main objective of this report was to generate DIAAS-like values to predict the PQ of a variety of plant and animal protein-containing ingredients potentially used for dog diet formulation. However, as DIAAS development in the human food industry relies on a reference IAA pattern based on the physiological IAA requirements of a specific population of interest (FAO, 2013), the secondary objective was to similarly assess PQ for dogs using a DIAAS equation adapted from the FAO (2013) wherein life stage-specific reference IAA patterns are applied. Fundamentally, the most appropriate IAA reference patterns to apply for dogs are based on MR estimates established by the NRC (2006) for adult dogs and growing puppies, as these represent physiological IAA requirements. Though, based on the pet food industry’s reliance on practical dietary IAA recommendations presented by AAFCO and FEDIAF as well as the recent literature reporting “DIAAS-like” values for dog food ingredients calculated using reference patterns based on IAA recommendations from these regulatory bodies (Oba et al., 2019; Do et al., 2020; Reilly et al., 2020a, 2020b, 2021; Gomez et al., 2021), we sought to assess the degree to which using these reference patterns may affect the PQ interpretation.

As expected, the choice of IAA reference pattern can greatly affect the resulting DIAAS-like value, and thus the subsequent interpretation of PQ. Although, while it appears as though a substantial amount of variability within these values stem from the selection of regulatory body, it has to be acknowledged that for the pet food industry to validly adopt PQ metrics such as DIAAS, it is critical that the same procedures are followed when utilizing these equations as are in the human food industry (FAO, 2013). Essentially, only reference patterns based on MR estimates established by the NRC for adult dogs and growing puppies should be applied considering they are the only IAA reference patterns based on physiological IAA requirements. While recommendations presented by AAFCO or FEDIAF are widely recognized and utilized throughout the pet food industry for commercial diet formulation purposes, these recommendations all, to an extent, take into account an assumed digestibility of mixed protein sources. Ultimately, while data presented herein should be interpreted prudently, particularly the DIAAS-like values generated using reference patterns associated with AAFCO or FEDIAF recommendations, this report should also be used to initiate a deeper conversation around: 1) how these recommendations are developed, 2) how they relate to the estimated physiological requirements presented by the NRC, 3) why they may differ between North American and European regulatory bodies and whether global synchronization is a necessary next step, and 4) whether the estimated physiological requirements and/or scaling factors used to account for factors like digestibility are indeed accurate.

The NRC is an advisory board organized by the United States National Academy of Sciences that has established an ad hoc nutrition committee designated to compile literature pertaining to the nutrient requirements for both dogs and cats. In 2000, this committee was tasked with revising the respective 1985 and 1986 publications on *Nutrient Requirements of Dogs* and *Nutrient Requirements of Cats* into one single report, while also thoroughly examining any relevant literature published since those editions were made available. The new report, *Nutrient Requirements of Dogs and Cats*, was published in 2006 and included updated estimations for requirements for all nutrients, including the IAA. In North America, regulatory recommendations for pet food formulation are defined by AAFCO, while FEDIAF establishes the regulatory guidelines in Europe. As discussed earlier, the AAFCO and FEDIAF dietary recommendations for IAA are essentially based on RA estimations from the NRC (2006) that have been scaled up by a factor of 1.25 to account for an assumed protein digestibility in a practical diet of 80%. Moreover, in the case of FEDIAF (2018) recommendations for adult dogs, an additional increase of 20% is applied to account for lower estimated energy requirements of household dogs compared to the average energy intake assumed by NRC (2006). Considering the multitude of factors that may affect diet protein digestibility and/or energy requirements of adult dogs, it brings into question the validity and accuracy of these scaling factors. Moreover, while NRC estimated requirements of IAA for growing dogs are based on extrapolated dose-response data from nitrogen balance and/or growth performance studies, the NRC states that “no individual dose-response peer-reviewed reports could be found for the minimal requirements of any of these amino acids (His, Ile, Leu, Lys, Phe, Thr, Trp, and Val) for dogs for maintenance” (2006). In fact, the estimated requirements of those aforementioned IAA for adult dogs at maintenance are based only on the lowest concentrations reported in one doctoral dissertation and one peer-reviewed report wherein dogs were fed low CP diets for an extended period of time and displayed no observable clinical signs of IAA deficiency (Ward, 1976; Sanderson et al., 2001). So, essentially, these MR estimates for adult dogs do not account for gut endogenous IAA losses, while those established for growing dogs do. Nevertheless, the NRC (2006) has indicated that no diet that meets the MR for CP and that is based on cereal grains, animal by-products, and plant proteins have been shown to be deficient in any of the IAA mentioned above. While that declaration may be true, that manner of thinking does not necessarily facilitate the advancement of our understanding of precise and sustainable pet nutrition. Furthermore, there has been a recent inundation of data generated using more advanced methodologies, such as indicator AA oxidation techniques, that challenge the accuracy of current regulatory recommendations with regard to dietary Phe, Trp, Met, Thr, and Lys requirements for adult dogs (Shoveller et al., 2017; Mansilla et al., 2018, 2020a, 2020b; Templeman et al., 2019; Harrison et al., 2020; Sutherland et al., 2020). Based on the progression of research in canine nutrition since 2006, it is increasingly clear that amendments to companion animal nutrient requirements are overdue, which would undoubtedly affect the estimation of ingredient PQ and the interpretation of this PQ metric as it relates the adequacy of protein ingredients and application of these data to develop complementary protein combinations.

With each DIAAS-like value, there is an associated LAA, unless the DIAAS-like value is greater than 100, in which case the LAA may not actually limit protein synthesis if fed in adequate amounts. As expected, SAA, Met, and Lys (plant ingredients in particular for the latter) were commonly identified LAA, which is potentially less problematic with regard to diet formulation considering that dietary supplementation of anhydrous DL-Met and Lys is commonplace in the pet food industry. However, attention should be drawn to the frequency at which Trp and Thr were identified as LAA, as these IAA are rarely supplemented in their anhydrous forms to commercial pet food, even though they are often considered for supplementation in animal agriculture. It is well known that complementary protein sources can be included in the diet to counteract potential IAA deficiencies and improve the PQ of the complete diet (Herreman et al., 2020). In fact, the FAO (2013) states that documentation of protein sources of greater PQ (higher DIAAS) with the intention of complementing proteins of lesser quality in mixed diets is one of the primary uses of this PQ model. This concept of complementary protein is vital in the human food industry, as humans typically consume a varied diet; however, ingredient complementarity is also fundamental in diet formulation for companion animals. With regard to diet formulation and/or production, the use of complementary protein sources helps to resolve difficulties related to protein ingredient sourcing and supply, a problem that has hampered the pet food industry globally, particularly in recent years (Hill, 2022). Moreover, as the trend of humanization continues to influence the pet food industry, the demand for products utilizing novel and alternative protein sources is increasing (Simonsen et al., 2014; Dodd et al., 2019; Schleicher et al., 2019), presenting an additional opportunity for the application of protein ingredient complementarity so as to ensure that diets formulated with less traditional protein sources provide sufficient levels of dietary nitrogen and all IAA. Nevertheless, it should be acknowledged that when using complementary sources to increase the inclusion of LAA such as Trp, which is often present in much lower concentrations relative to its requirement than other IAA (Templeman et al., 2020), the attempt to meet the Trp requirement may result in other IAA being supplied in excess. Ultimately, if we are to ensure that the industry remains sustainable, resources must be directed toward identifying ways we can approach diet formulation from an “ideal protein” or “precision protein” perspective, whether it be with anhydrous AA supplementation and/or complementary protein sources.

Ultimately, it was hypothesized that applying IAA reference patterns based on IAA requirements or recommendations for growth would result in DIAAS-like values that would exceed those generated when applying IAA patterns for maintenance; however, while this was observed when NRC and FEDIAF reference patterns were applied, it was not always the case when applying AAFCO reference patterns. This outcome was a reflection of the DIAAS-like values presented herein being generated using reference IAA patterns on an mg IAA/g CP basis. The practical IAA recommendations proposed by AAFCO to support GR were, in most cases, nearly 2-fold that of maintenance, yet the CP recommendations only marginally different between the two life stages. Essentially, these data underpin the stark differences in proposed nutrient guidelines across scientific and regulatory bodies, even with a dearth of actual empirical estimates of biological requirements. This clearly highlights the need for research on protein and IAA requirements under different conditions as well as a unified regulatory approach to facilitate the global pet food market.

The authors do acknowledge that the present study has limitations aside from those previously discussed regarding the use of AAFCO/FEDIAF IAA reference patterns, including that there were characteristics of these ingredients (e.g., degree of processing, presence of anti-nutritional factors) that could not be captured in this report, but that could affect the interpretation of PQ. However, due to the sheer quantity of data, some definitive boundaries had to be set. Moreover, it is assumed that some of these effects were inherently captured in the digestibility coefficients used for the PQ equations. There has been an increased interest in less processed ingredients as well as plant protein ingredients in the pet food industry, largely driven by the need to satisfy consumer demand (Simonsen et al., 2014; Dodd et al., 2019; Schleicher et al., 2019). As such, future research is necessary to investigate how various commonly used methods of ingredient (e.g., rendering, drying) or diet processing (e.g., extrusion and freeze–drying) as well as the presence/activity of anti-nutritional factors (e.g., protease inhibitors, lectins, and tannins) may influence these metrics of PQ. This is of importance as processing conditions (e.g., cooking temperature) and anti-nutritional factor content have been shown to affect the quality of protein-containing ingredients, with the latter predominantly affecting plant ingredients (Johnson et al., 1998; Opstvedt et al., 2003; Sarwar et al., 2012; Hodgkinson et al., 2018). Furthermore, while processing conditions, such as heat treatment, can induce deleterious molecular alterations to proteins (e.g., Maillard reaction; González-Vega et al., 2011) rendering them less digestible, these same conditions may be necessary to reduce the concentrations of anti-nutritional factors (Purushotham et al., 2007). By improving our knowledge of how these factors may influence nutrient availability/ingredient quality, we can help to meet the demands of the pet owners for diets formulated with less processed ingredients or with a greater contribution from plant ingredients while also ensuring that changes in PQ and nutrient availability are accounted for when considering the processing conditions of diet production. Finally, it should be acknowledged that this report is based solely upon publicly available literature and, as such, the outcomes presented herein are only a reflection of the amount and type of ingredient data available.

## Conclusions and Implications

The current work provides DIAAS-like values for numerous animal and plant ingredients potentially used in the pet food industry. The authors acknowledge that for the pet food industry to align with the human food industry with regards to the assessment of ingredient PQ, that only reference patterns based on MR estimate established by the NRC should be applied as these are based on an adult or growing dog’s physiological IAA requirements. However, the reliance of the pet food industry on IAA and CP recommendations presented by AAFCO and FEDIAF as well as the recent arrival of literature assessing PQ using reference patterns based on these regulatory body recommendations encouraged us to present and discuss how the selection of reference pattern may affect PQ interpretation. Ultimately, while future studies assessing PQ of ingredients or complete commercial diets should utilize MR estimates presented by the NRC, difference in DIAAS-like values for ingredients when using AAFCO and FEDIAF recommendations underpin the different regulatory approaches to establishing dietary nutrient recommendations that exist globally and suggest that harmonization these recommendations may be valuable, especially with consideration of the globalization of pet food companies.

## Supplementary Material

skac279_suppl_Supplementary_Appendix

skac279_suppl_Supplementary_Materials

## Abbreviations

AA: amino acid
AAA: aromatic amino acids (Phe + Tyr)
AAFCO: Association of American Feed Control Officials
AM: adult maintenance
CP: crude protein
DIAAS: digestible indispensable amino acid score
DDGS: distillers dried grain with solubles
DM: dry matter
EG: early growth
FAO: Food and Agriculture Organization
FEDIAF: European Pet Food Industry Federation
GR: growth and reproduction
IAA: indispensable amino acid
LAA: limiting amino acid
LG: late growth
MR: minimal requirement
NRC: National Research Council
PDCAAS: protein digestibility-corrected amino acid score
PQ: protein quality
RA: recommended allowance
SAA: sulfur amino acids (Met + Cys)

## References

[CIT0001] Almeida, F. N., G. I. Petersen, and H. H. Stein. 2011. Digestibility of amino acids in corn, corn coproducts, and bakery meal fed to growing pigs. J. Anim. Sci. 89:4109–4115. doi:10.2527/jas.2011-4143 21821809

[CIT0002] Association of American Feed Control Officials. 2016. AAFCO manual. West Lafayette (IN): AAFCO Inc.

[CIT0003] Bailey, H. M., J. K. Mathai, E. P. Berg, and H. H. Stein. 2020. Pork products have digestible indispensable amino acid scores (DIAAS) that are greater than 100 when determined in pigs, but processing does not always increase DIAAS. J. Nutr. 150:475–482. doi:10.1093/jn/nxz284 31758187

[CIT0004] Batterham, E. S. 1992. Availability and utilization of amino acids for growing pigs. Nutr. Res. Rev. 5:1–18. doi:10.1079/NRR19920004 19094310

[CIT0005] Cervantes-Pahm, S. K., Y. Liu, and H. H. Stein. 2014. Digestible indispensable amino acid score and digestible amino acids in eight cereal grains. Br. J. Nutr. 111:1663–1672. doi:10.1017/S0007114513004273 24480298

[CIT0006] Columbus, D. A., H. Lapierre, J. K. Htoo, and C. F. de Lange. 2014. Nonprotein nitrogen is absorbed from the large intestine and increases nitrogen balance in growing pigs fed a valine-limiting diet. J. Nutr. 144:614–620. doi:10.3945/jn.113.187070 24647394

[CIT0007] Cotten, B., D. Ragland, J. E. Thomson, and O. Adeola. 2016. Amino acid digestibility of plant protein feed ingredients for growing pigs. J. Anim. Sci. 94:1073–1082. doi:10.2527/jas.2015-9662 27065269

[CIT0008] Cramer, K. R., M. W. Greenwood, J. S. Moritz, R. S. Beyer, and C. M. Parsons. 2007. Protein quality of various raw and rendered by-product meals commonly incorporated into companion animal diets. J. Anim. Sci. 85:3285–3293. doi:10.2527/jas.2006-225 17609474

[CIT0009] CVB Feed Table. 2016. Chemical composition and nutritional values of feedstuffs. Wageningen (The Netherlands): Federatie Nederlandse Diervoederketen.

[CIT0010] Darragh, A. J., P. D. Cranwell, and P. J. Moughan. 1994. Absorption of lysine and methionine from the proximal colon of the piglet. Br. J. Nutr. 71:739–752. doi:10.1079/bjn19940181 8054329

[CIT0011] Deglaire, A., C. Bos, D. Tomé, and P. J. Moughan. 2009. Ileal digestibility of dietary protein in the growing pig and adult human. Br. J. Nutr. 102:1752–1759. doi:10.1017/S0007114509991267 19706206

[CIT0012] Do, S., L. Koutsos, P. L. Utterback, C. M. Parsons, M. de Godoy, and K. S. Swanson. 2020. Nutrient and AA digestibility of black soldier fly larvae differing in age using the precision-fed cecectomized rooster assay. J. Anim. Sci. 98:skz363. doi:10.1093/jas/skz363 31781760 PMC6978894

[CIT0013] Dodd, S. A. S., N. J. Cave, J. L. Adolphe, A. K. Shoveller, and A. Verbrugghe. 2019. Plant-based (vegan) diets for pets: a survey of pet owner attitudes and feeding practices. PLoS One 14:e0210806. doi:10.1371/journal.pone.0210806 30645644 PMC6333351

[CIT0014] European Pet Food Industry Federation. 2018. Nutritional guidelines for complete and complementary pet food for cats and dogs. Bruxelles (Belgium): FEDIAF Inc.

[CIT0015] Faber, T. A., P. J. Bechtel, D. C. Hernot, C. M. Parsons, K. S. Swanson, S. Smiley, and G. C. Fahey Jr. 2010. Protein digestibility evaluations of meat and fish substrates using laboratory, avian, and ileally cannulated dog assays. J. Anim. Sci. 88:1421–1432. doi:10.2527/jas.2009-2140 20023140

[CIT0016] Food and Agriculture Organization. 2013. Dietary protein quality evaluation in human nutrition. Report of an FAO expert consultation. FAO Food Nutrition Paper; p. 92. 26369006

[CIT0017] Food and Agriculture Organization/World Health Organization-United Nations. 1985. Energy and protein requirements. Report of a joint FAO/WHO/UNU expert consultation, WHO Tech. Rep. Ser. Pap; p. 724. 3937340

[CIT0018] Fuller, M. F., and P. J. Reeds. 1998. Nitrogen cycling in the gut. Annu. Rev. Nutr. 18:385–411. doi:10.1146/annurev.nutr.18.1.385 9706230

[CIT0019] Gomez, V. A., P. L. Utterback, C. M. Parsons, M. de Godoy, and H. Russell. 2021. Evaluation of macronutrient composition and amino acid digestibility of select novel dietary proteins for use in canine and feline nutrition. J. Anim. Sci. 99:57 (Abstr.). doi:10.1093/jas/skab235.102

[CIT0020] González-Vega, J. C., B. G. Kim, J. K. Htoo, A. Lemme, and H. H. Stein. 2011. Amino acid digestibility in heated soybean meal fed to growing pigs. J. Anim. Sci. 89:3617–3625. doi:10.2527/jas.2010-3465 21742940

[CIT0021] Gottlob, R. O., J. M. DeRouchey, M. D. Tokach, R. D. Goodband, S. S. Dritz, J. L. Nelssen, C. W. Hastad, and D. A. Knabe. 2006. Amino acid and energy digestibility of protein sources for growing pigs. J. Anim. Sci. 84:1396–1402. doi:10.2527/2006.8461396x 16699096

[CIT0022] Grosjean, F., C. Jondreville, I. Williatte-Hazouard, F. Skiba, B. Carrouée, and F. Gâtel. 2000. Ileal digestibility of protein and amino acids of feed peas with different trypsin inhibitor activity in pigs. Can. J. Anim. Sci. 80:643–652. doi:10.4141/a99-075

[CIT0023] Harrison, M. D., M. E. Jackson, D. G. McLaren, C. M. Parsons, and D. A. Knabe. 1990. A direct comparison of true amino acid digestibility determined with poultry and apparent amino acid digestibility determined with swine. Poult. Sci. 69:38.

[CIT0024] Harrison, M., G. Thomas, M. Gilham, K. Gray, A. Colyer, and D. Allaway. 2020. Short-term determination and long-term evaluation of the dietary methionine requirement in adult dogs. Br. J. Nutr. 26:1–12. doi:10.1017/S0007114520000690 32100649

[CIT0025] Hendriks, W. H., E. J. Baker, and G. Bosch. 2015. Protein and amino acid bioavailability estimates for canine foods. J. Anim. Sci. 93:4788–4795. doi:10.2527/jas.2015-9231 26523572

[CIT0026] Herreman, L., P. Nommensen, B. Pennings, and M. C. Laus. 2020. Comprehensive overview of the quality of plant- and animal-sourced proteins based on the digestible indispensable amino acid score. Food Sci. Nutr. 8:5379–5391. doi:10.1002/fsn3.1809 33133540 PMC7590266

[CIT0027] Hill, M. R. 2022. Evaluating the supply chain of animal protein-based pet food ingredients and international trade of pet food. MSc Diss. Manhattan (NY): Kansas State University.

[CIT0028] Hodgkinson, A. J., O. A. M. Wallace, I. Boggs, M. Broadhurts, and C. G. Prosser. 2018. Gastric digestion of cow and goat milk: impact of infant and young child *in vitro* digestion conditions. Food Chem. 245:275–281. doi:10.1016/j.foodchem.2017.10.028 29287371

[CIT0029] Johnson, M. L., C. M. Parsons, G. C. Fahey, Jr., N. R. Merchen, and C. G. Aldrich. 1998. Effects of species raw material source, ash content, and processing temperature on amino acid digestibility of animal by-product meals by cecectomized roosters and ileally cannulated dogs. J. Anim. Sci. 76:1112–1122. doi:10.2527/1998.7641112x 9581935

[CIT0030] Kerr, B. J., P. E. Urriola, R. Jha, J. E. Thomson, S. M. Curry, and G. C. Shurson. 2019. Amino acid composition and digestible amino acid content in animal protein by-product meals fed to growing pigs. J. Anim. Sci. 97:4540–4547. doi:10.1093/jas/skz294 31587052 PMC6827409

[CIT0031] Kong, C., and O. Adeola. 2014. Evaluation of amino acid and energy utilization in feedstuff for swine and poultry diets. Asian-Australas. J. Anim. Sci. 27:917. doi:10.5713/ajas.2014.r.02 25050031 PMC4093562

[CIT0032] Lee, S. A., J. Y. Ahn, A. R. Son, and B. G. Kim. 2020. Standardized ileal digestibility of amino acids in cereal grains and co-products in growing pigs. Asian-Australas. J. Anim. Sci. 33:1148–1155. doi:10.5713/ajas.19.0449 32054200 PMC7322653

[CIT0033] Leme, B. B., N. Sakomura, G. S. Viana, L. Soares, M. C. Melaré, M. J. K. Oliveira, and C. F. M. Mansano. 2019. Amino acid digestibility of feed ingredients in cecectomized adult roosters. Brazil. J. Poult. Sci. 21. doi:10.1590/1806-9061-2018-0924

[CIT0034] Mansilla, W. D., L. Fortener, A. Gorman, and A. K. Shoveller. 2018. Dietary phenylalanine requirements are similar in small, medium, and large breed adult dogs using the indicator amino acid oxidation technique. J. Anim. Sci. 96:3112–3120. doi:10.1093/jas/sky208 29846616 PMC6095264

[CIT0035] Mansilla, W. D., J. R. Templeman, L. Fortener, and A. K. Shoveller. 2020a. Minimum dietary methionine requirements in Miniature Dachshund, Beagle, and Labrador Retriever adult dogs using the indicator amino acid oxidation technique. J. Anim. Sci. 98:skaa324. doi:10.1093/jas/skaa324 33011778 PMC7751151

[CIT0036] Mansilla, W. D., J. R. Templeman, L. Fortener, and A. K. Shoveller. 2020b. Adult dogs of different breed sizes have similar threonine requirements as determined by the indicator amino acid oxidation technique. J. Anim. Sci. 98:skaa066. doi:10.1093/jas/skaa066 32108874 PMC7085255

[CIT0037] Mathai, J. K., Y. Liu, and H. H. Stein. 2017. Values for digestible indispensable amino acid scores (DIAAS) for some dairy and plant proteins may better describe protein quality than values calculated using the concept for protein digestibility-corrected amino acid scores (PDCAAS). Br. J. Nutr. 117:490–499. doi:10.1017/S0007114517000125 28382889

[CIT0038] Morris, J. G., and Q. R. Rogers. 1994. Assessment of the nutritional adequacy of pet foods through the life cycle. J. Nutr. 124:2520S–2534S. doi:10.1093/jn/124.suppl_12.2520S 7996231

[CIT0039] Moughan, P. J., and A. Rowan. 1989. The pig as a model animal for human nutrition research. N. Z. Nutr. Soc. 14:116–123.

[CIT0040] Murray, S. M., R. P. Avinash, G. C. Fahey Jr., N. M. Merchen, and D. M. Hughes. 1997. Raw and rendered animal by-products as ingredients in dog diets. J. Nutr. 128:2812S–2815S. 10.2527/1997.7592497x 9868275

[CIT0041] National Research Council. 2006. Nutrient requirements of dogs and cats. 2nd rev. ed. Washington (DC): National Academic Press.

[CIT0042] National Research Council. 2012. Nutrient requirements of swine. 11th rev. ed. Washington (DC): National Academic Press.

[CIT0043] Nosworthy, M. G., and J. D. House. 2017. Factors influencing the quality of dietary proteins: implications for pulses. Cereal Chem. 94:49–57. doi:10.1094/CCHEM-04-16-0104-FI

[CIT0044] Oba, P. M., P. L. Utterback, C. M. Parsons, and K. S. Swanson. 2019. True nutrient and amino acid digestibility of dog foods made with human-grade ingredients using the precision-fed cecectomized rooster assay. Transl. Anim. Sci. 4:442–451. doi:10.1093/tas/txz175 32705002 PMC6994059

[CIT0045] Opstvedt, J., E. Nygård, T. A. Samuelsen, G. Venturini, U. Luzzana, and H. Mundheim. 2003. Effect on protein digestibility of different processing conditions in the production of fish meal and fish feed. J. Sci. Food Agric. 83:775–782. doi:10.1186/1471-2164-13-363

[CIT0046] Park, C. S., V. D. Naranjo, J. K. Htoo, and O. Adeola. 2020. Comparative amino acid digestibility between broiler chickens and pigs fed different poultry by-products and meat and bone meal. J. Anim. Sci. 98:skaa223. doi:10.1093/jas/skaa223 32667675 PMC7455265

[CIT0047] Prolla, I. R. D., M. Rafii, G. Courtney-Martin, R. Elango, L. P. da Silva, R. O. Ball, and P. B. Pencharz. 2013. Lysine from cooked white rice consumed by healthy young men is highly metabolically available when assessed using the indicator amino acid oxidation technique. J. Nutr. 143:302–306. doi:10.3945/jn.112.166728 23325920

[CIT0048] Purushotham, B., P. M. Radhakrishna, and B. S. Sherigara. 2007. Effects of steam conditioning and extrusion temperature on some antinutritional factors of soyabean (Glycine max) for pet food applications. Am. J. Anim. Vet. Sci. 2:1–5. doi:10.3844/ajavsp.2007.1.5

[CIT0049] Reilly, L. M., P. C. von Schaumburg, J. M. Hoke, G. M. Davenport, P. L. Utterback, C. M. Parsons, and M. de Godoy. 2020a. Use of precision-fed cecectomized rooster assay and digestible indispensable amino acid scores to characterize plant- and yeast-concentrated proteins for inclusion in canine and feline diets. Transl. Anim. Sci. 4:txaa133. doi:10.1093/tas/txaa133 32832856 PMC7433924

[CIT0050] Reilly, L. M., P. C. von Schaumburg, J. M. Hoke, G. M. Davenport, P. L. Utterback, C. M. Parsons, and M. de Godoy. 2020b. Macronutrient composition, true metabolizable energy and amino acid digestibility, and indispensable amino acid scoring of pulse ingredients for use in canine and feline diets. J. Anim. Sci. 98:1–8. doi:10.1093/jas/skaa149 PMC7265953 32484865

[CIT0051] Reilly, L. M., P. C. von Schaumburg, J. M. Hoke, G. M. Davenport, P. L. Utterback, C. M. Parsons, and M. de Godoy. 2021. Use of precision-fed cecectomized rooster assay to determine standardized amino acid digestibility, true metabolizable energy content, and digestible indispensable amino acid scores of plant-based protein by-products used in canine and feline diets. Transl. Anim. Sci. 5:1–9. doi:10.1093/tas/txab025 PMC8244986 34222818

[CIT0052] Rojas, O. J. and H. H. Stein. 2012. Nutritional value of animal proteins fed to pigs. Proc. Midwest Swine Nutr. Conf., Indianapolis, IN. p. 9–24.

[CIT0053] Rowan, A. M., P. J. Moughan, M. Wilson, K. Maher, and C. Tasman-Jones. 1994. Comparison of the ileal and faecal digestibility of dietary amino acids in adult humans and evaluation of the pig as a model animal for digestion studies in man. Br. J. Nutr. 71:29–42. doi:10.1079/bjn19940108 8312239

[CIT0054] Sanderson, S. L., K. L. Gross, P. N. Ogburn, C. Calvert, G. Jacobs, S. R. Lowry, K. A. Bird, L. A. Koehler, and L. L. Swanson. 2001. Effects of dietary fat and L-carnitine on plasma and whole blood taurine concentrations and cardiac function in healthy dogs fed protein-restricted diets. Am. J. Vet. Res. 62:1616–1623. doi:10.2460/ajvr.2001.62.1616 11592329

[CIT0055] Sarwar, G. G., X. C. Wu, and K. A. Cockell. 2012. Impact of antinutritional factors in food proteins on the digestibility of protein and the bioavailability of amino acids and on protein quality. Br. J. Nutr. 108:S315–S332. doi:10.1017/S0007114512002371 23107545

[CIT0056] Sauvant, D., J. M. Perez, and G. Tran. 2004. Tables of composition and nutritive value of feed materials: pigs, poultry, cattle, sheep, goats, rabbits, horses and fish. Wageningen (The Netherlands): Wageningen Academic Publishers.

[CIT0057] Schleicher, M., S. B. Cash, and L. M. Freeman. 2019. Determinants of pet food purchasing decisions. Can. Vet. J. 60:644–650. 31156266 PMC6515811

[CIT0058] Shoveller, A. K., J. J. Danelon, J. L. Atkinson, G. M. Davenport, R. O. Ball, and P. B. Pencharz. 2017. Calibration and validation of a carbon oxidation system and determination of the bicarbonate retention factor and the dietary phenylalanine requirement, in the presence of excess tyrosine, of adult, female, mixed-breed dogs. J. Anim. Sci. 95:2917–2927. doi:10.2527/jas.2017.1535 28727110

[CIT0059] Sibbald, I. R. 1979. A bioassay for available amino acids and true metabolizable energy in feeding stuffs. Poult. Sci. 58:668–673. doi:10.3382/ps.0580668

[CIT0060] Simonsen, J. E., G. M. Faskeno, and J. M. Lillywhite. 2014. The value-added dog food market: do dog owners prefer natural or organic dog foods? J. Agric. Sci. 6:86–97. doi:10.5539/JAS.V6N6P86

[CIT0061] Stein, H. H., M. F. Fuller, P. J. Moughan, B. Sève, R. Mosenthin, A. J. M. Jansman, J. A. Fernandez, and C. F. M. de Lange. 2007a. Definition of apparent, true, and standardized ileal digestibility of amino acids in pigs. Livest. Sci. 109:282–285. doi:10.1016/j.livsci.2007.01.019

[CIT0062] Stein, H. H., B. Sève, M. F. Fuller, P. J. Moughan, and C. F. M. de Lange. 2007b. Invited review: amino acid bioavailability and digestibility in pig feed ingredients: terminology and application. J. Anim. Sci. 85:172–180. doi:10.2527/jas.2005-742 17179553

[CIT0063] Sutherland, K., W. D. Mansilla, L. Fortener, and A. K. Shoveller. 2020. Lysine requirements in small, medium, and large breed adult dogs using the indicator amino acid oxidation technique. Transl. Anim. Sci. 4:txaa082. doi:10.1093/tas/txaa082 32734145 PMC7381836

[CIT0064] Templeman, J. R., W. D. Mansilla, L. Fortener, and A. K. Shoveller. 2019. Tryptophan requirements in small, medium, and large breed adult dogs using the indicator amino acid oxidation technique. J. Anim. Sci. 97:3274–3285. doi:10.1093/jas/skz142 31363781 PMC6667247

[CIT0065] Templeman, J. R., E. Thornton, C. Cargo-Froom, E. J. Squires, K. S. Swanson, and A. K. Shoveller. 2020. Effects of incremental exercise and dietary tryptophan supplementation on the amino acid metabolism, serotonin status, stool quality, fecal metabolites, and body composition of mid-distance training sled dogs. J. Anim. Sci. 98:skaa128. doi:10.1093/jas/skaa128 32315027 PMC7228677

[CIT0066] Ward, J. 1976. The amino acid requirements of the adult dog [Ph.D. dissertation]. Cambridge (UK): Wolfson College, University of Cambridge.

[CIT0067] Woyengo, T. A., J. M. Heo, Y. L. Yin, and C. M. Nyachoti. 2015. Standardized and true ileal amino acid digestibilities in field pea and pea protein isolate fed to growing pigs. Anim. Feed Sci. Technol. 207:19s6–203. doi:10.1016/j.anifeedsci.2015.06.008

[CIT0068] Zhang, C. X., K. K. Huang, L. Wang, K. Song, L. Zhang, and P. Li. 2015. Apparent digestibility coefficients and amino acid availability of common protein ingredients in the diets of bullfrog, *Rana (Lithobates) catesbeiana*. Aquaculture 437:38–45. doi:10.1ss016/j.aquaculture.2014.11.015

