# Supplementary material for: Digestible indispensable amino acid scores of animal and plant ingredients potentially used in dog diet formulation: how this protein quality metric is affected by ingredient characteristics and reference amino acid profile
Source: J Anim Sci. 2022 Aug 27;100(11):skac279. doi: 10.1093/jas/skac279 (PMC9624197; doi:10.1093/jas/skac279)
Supplement: skac279_suppl_Supplementary_Appendix [file skac279_suppl_supplementary_appendix.docx]

**APPENDICES**

**Appendix 1.** Detailed ingredient classification.

In total, 30 animal (75 unique inputs from literature) and 27 plant ingredients (94 unique inputs from literature) satisfied all inclusion criteria and were captured in the final data set (Tables 1 and Table 2 for animal and plant ingredients, respectively). Based on AAFCO (2016) Official Common and Usual Names and Definitions of Feed Ingredients, all animal ingredients were *collectively* classified as ‘animal protein products’, while plant ingredients were *collectively* classified as either a ‘plant protein product’ (14 ingredients, 48 unique inputs), a ‘processed grain by-product’ (6 ingredients, 16 unique inputs), or a ‘grain product’ (7 ingredients, 30 unique inputs).

Ingredients were further categorized based on *broad* and *specific* AAFCO classifications (AAFCO, 2016). Animal ingredients were *broadly* classified as either an ‘animal product’ (22 ingredients, 55 unique inputs), a ‘milk product’ (5 ingredients, 14 unique inputs), or a ‘marine product’ (3 ingredients, 6 unique inputs), and then *specifically* classified as either ‘animal by-product meal’, ‘blood meal’, ‘egg product’, ‘hydrolyzed poultry feathers’, ‘meat’, ‘meat and bone meal’, ‘meat meal’, ‘poultry by-product meal’ and ‘poultry meal’ (animal products), ‘casein’, ‘dried milk’, ‘dried skimmed milk’, ‘dried whey concentrate’ and ‘dried whey’ (milk products), or ‘fish meal’ and ‘meat’ (marine products; Table 1).

Plant ingredients were *broadly* classified as either a ‘barley product’ (1 ingredient, 4 unique inputs), a ‘cottonseed product’ (1 ingredient, 2 unique inputs), a ‘distillers product’ (2 ingredients, 4 unique inputs), ‘grain sorghum’ (1 ingredient, 4 unique inputs), a ‘maize product’ (3 ingredients, 14 unique inputs), a ‘miscellaneous product’ (4 ingredients, 18 unique inputs), an ‘oat product’ (2 ingredients, 6 unique inputs), an ‘other oilseed product’ (4 ingredients, 9 unique inputs), a ‘rice product’ (2 ingredients, 6 unique inputs), a ‘rye product’ (1 ingredient, 4 unique inputs), a ‘soybean product’ (2 ingredients, 8 unique inputs), a ‘wheat product’ (3 ingredients, 12 unique inputs), or a ‘yeast product’ (1 ingredient, 3 unique inputs). These plant ingredients were then *specifically* classified as either ‘barley grain’ (captured as ‘barley products’), ‘cottonseed meal’ (captured as ‘cottonseed products’), ‘dried distillers grain with solubles’ (captured as ‘distillers products’), ‘grain sorghum’ (captured as ‘grain sorghum products’), ‘corn germ meal’, ‘corn gluten meal’ and ‘corn grain’ (captured as ‘maize products’), ‘dried bean’, ‘dried pea’ (common name) and ‘potato protein’ (captured as ‘miscellaneous products’), ‘oat groats’ and ‘oat grain’ (captured as ‘oat products’), ‘canola meal’, ‘linseed meal’, ‘peanut meal’ and sunflower meal’ (captured as ‘other oilseed products’), ‘rice bran’ and ‘rice grain’ (captured as ‘rice products’), ‘rye grain’ (captured as ‘rye products’), ‘soybean meal’ and ‘soy protein concentrate’ (captured as ‘soybean products’), ‘wheat bran’, ‘wheat middlings’ and ‘wheat grain’ (captured as ‘wheat products’), or ‘brewers dried yeast’ (captured as ‘yeast products’; Table 2).

**Appendix 2.** Detailed overview of DIAAS-like values and CP content results for each *broad* AAFCO ingredient category of the plant ingredients.

When using NRC and FEDIAF AM reference patterns, ‘rice products’ produced the greatest DIAAS-like values, while ‘cottonseed products’, ‘maize products’, ‘miscellaneous products’, and ‘yeast products’ produced the lowest DIAAS-like values (P *≤*0.05; Supplementary Table 2). When using NRC EG and LG and FEDIAF EG reference patterns, ‘rice products’ and ‘soybean products’ produced the greatest DIAAS-like values, while ‘distillers products’ produced the lowest (P *≤*0.05; Supplementary Table 2). When using AAFCO AM reference patterns, ‘barley products’ and ‘oat products’ produced the greatest DIAAS-like values, while ‘distillers products’ produced the lowest (P *≤*0.05; Supplementary Table 2). By using AAFCO GR as a reference pattern, ‘soybean products’ produced the greatest DIAAS-like values, while ‘distillers products’ produced the lowest (P *≤*0.05; Supplementary Table 2). Last, when using FEDIAF LG as a reference pattern, ‘soybean products’ produced the greatest DIAAS-like values, while ‘distillers products’ and ‘maize products’ produced the lowest (P *≤*0.05; Supplementary Table 2).

In addition, ‘soybean products’, ‘miscellaneous products’, and ‘yeast products’ had the greatest CP content (% DM basis), while ‘barley products’, ‘oat products’, ‘rice products’, ‘rye products’, and ‘wheat products’ had the lowest CP content (P *≤*0.05; Supplementary Table 2).

**Appendix 3.** Detailed overview of DIAAS-like values and CP content results for each *collective* AAFCO ingredient category.

For NRC EG and LG, AAFGO GR, and FEDIAF EG and LG reference patterns, there were no differences observed between DIAAS-like values of ‘plant protein products’, ‘processed grain by-products’ and ‘grain products’, and in no scenario did DIAAS-like values differ between ‘animal protein products’ and ‘plant protein products’ (P > 0.05; Supplementary Table 3). For NRC, AAFCO, and FEDIAF AM reference patterns, ‘grain products’ produced the greatest DIAAS, while ‘plant protein products’ produced the lowest DIAAS (P *≤*0.05; Supplementary Table 3); however, in none of those scenarios did DIAAS differ between ‘animal protein products’ and ‘plant protein products’ (P > 0.05; Supplementary Table 3).

Moreover, ‘plant protein products’ had the greatest CP content (% DM basis), while ‘grain products’ had the lowest (P *≤*0.05; Supplementary Table 3), and the animal ingredient collective AAFCO category of ‘animal protein products’ had greater CP content than ‘plant protein products’ (P *≤*0.05; Supplementary Table 3).
