# Supplementary material for: Digestible indispensable amino acid scores of animal and plant ingredients potentially used in dog diet formulation: how this protein quality metric is affected by ingredient characteristics and reference amino acid profile
Source: J Anim Sci. 2022 Aug 27;100(11):skac279. doi: 10.1093/jas/skac279 (PMC9624197; doi:10.1093/jas/skac279)
Supplement: skac279_suppl_Supplementary_Materials [file skac279_suppl_supplementary_materials.docx]

**SUPPLEMENTARY MATERIALS**

**Supplementary Table 1.** Least-squares means (± SEM) for digestible indispensable amino acid scores (DIAAS)-like values and crude protein content (CP, % dry matter basis) of animal ingredients categorized based on broad AAFCO Official Common and Usual Names and Definitions of Feed Ingredients as determined using NRC, AAFCO, or FEDIAF^1^ amino acid requirements or recommendations at each discrete life stage as a reference pattern.

| IAA reference pattern | | Variable^2^ | Broad AAFCO classification | | | SEM | *P*-value |
| --- | --- | --- | --- | --- | --- | --- | --- |
|  |  |  | Animal product | Marine product | Milk product |  |  |
| NRC | AM^3^ | DIAAS-like | 30.56^b^ | 47.43^a^ | 53.40^a^ | 4.31 | <0.01 |
|  | EG |  | 55.75^b^ | 85.80^a^ | 93.46^a^ | 9.11 | <0.01 |
|  | LG |  | 56.77^a^ | 86.26^b^ | 88.81^b^ | 9.20 | <0.02 |
| AAFCO | AM | DIAAS-like | 53.19^b^ | 85.05^a^ | 91.23^a^ | 7.76 | <0.01 |
|  | GR |  | 49.72^b^ | 73.25^a^ | 72.43^a^ | 7.16 | <0.01 |
| FEDIAF | AM | DIAAS-like | 46.57^b^ | 73.69^a^ | 80.84^a^ | 6.64 | <0.01 |
|  | EG |  | 61.67^b^ | 93.72^a^ | 101.01^a^ | 10.01 | <0.01 |
|  | LG |  | 59.07^b^ | 84.17^a^ | 91.90^a^ | 9.24 | <0.01 |
| - | | CP | 69.28^a^ | 68.96^a^ | 47.42^b^ | 4.74 | <0.01 |

^1^NRC, National Research Council Nutrient Requirements of Dogs and Cats (2006); AAFCO, Association of American Feed Control Officials (2016); FEDIAF, European Pet Food Industry Federation Nutritional Guidelines for Complete and Complementary Pet Foods for Cats and Dogs (2018).
^2^DIAAS-like, digestible indispensable amino acid score-like values; CP, crude protein content (%, dry matter basis).
^3^AM, adult maintenance; EG, early growth (NRC, 4-14 weeks of age; FEDIAF, less than 14 weeks of age); LG, late growth (NRC and FEDAF, greater than 14 weeks of age), GR, growth and reproduction (AAFCO only).
^a,b^ Values in a row with different superscript are significantly different (*P* ≤ 0.05).

**Supplementary Table 2.** Least-squares means (± SEM) for digestible indispensable amino acid scores (DIAAS)-like values and crude protein content (CP, % dry matter basis) of plant ingredients categorized based on broad AAFCO Official Common and Usual Names and Definitions of Feed Ingredients as determined using NRC, AAFCO, or FEDIAF^1^ amino acid requirements or recommendations at each discrete life stage as a reference pattern.

| IAA reference pattern | | Variable^2^ | Broad AAFCO classification | | | | | | | | | | | | | SEM | *P*-value |
| --- | --- | --- | --- | --- | --- | --- | --- | --- | --- | --- | --- | --- | --- | --- | --- | --- | --- |
|  |  |  | Barley product | Cottonseed product | Distillers product | Grain sorghum | Maize product | Miscellaneous product | Oat product | Other oilseed product | Rice product | Rye product | Soybean product | Wheat product | Yeast product |  |  |
| NRC | AM^3^ | DIAAS-like | 42.42^ab^ | 26.02^b^ | 36.42^ab^ | 39.37^ab^ | 33.72^b^ | 27.30^b^ | 43.11^ab^ | 39.62^ab^ | 51.22^a^ | 34.31^ab^ | 36.75^ab^ | 39.69^ab^ | 29.01^b^ | 6.20 | <0.01 |
|  | EG |  | 68.90^ab^ | 49.10^ab^ | 34.52^b^ | 41.63^ab^ | 44.16^ab^ | 54.92^ab^ | 68.89^ab^ | 63.25^ab^ | 73.46^a^ | 55.19^ab^ | 71.12^a^ | 61.77^ab^ | 50.46^ab^ | 11.24 | <0.01 |
|  | LG |  | 68.65^ab^ | 49.51^ab^ | 33.38^b^ | 40.20^ab^ | 43.49^ab^ | 56.13^ab^ | 68.91^a^ | 63.94^ab^ | 73.76^a^ | 55.57^ab^ | 72.95^a^ | 61.51^ab^ | 51.00^ab^ | 11.39 | <0.01 |
| AAFCO | AM | DIAAS-like | 74.41^a^ | 45.89^ab^ | 38.53^b^ | 46.60^ab^ | 49.70^ab^ | 48.57^ab^ | 74.69^a^ | 68.89^a^ | 85.73^ab^ | 60.95^ab^ | 63.96^ab^ | 66.21^ab^ | 51.73^ab^ | 10.40 | <0.01 |
|  | GR |  | 53.66^ab^ | 39.98^ab^ | 33.78^b^ | 40.82^ab^ | 45.05^ab^ | 54.37^ab^ | 55.87^ab^ | 53.58^ab^ | 58.97^ab^ | 43.33^ab^ | 69.10^a^ | 50.54^ab^ | 52.03^ab^ | 9.97 | <0.01 |
| FEDIAF | AM | DIAAS-like | 63.11^ab^ | 38.87^b^ | 56.49^ab^ | 58.05^ab^ | 49.16^b^ | 40.96^b^ | 63.53^ab^ | 59.91^ab^ | 76.21^a^ | 50.84^ab^ | 54.84^ab^ | 58.84^ab^ | 43.57^b^ | 9.47 | <0.01 |
|  | EG |  | 76.42^ab^ | 54.75^ab^ | 38.02^b^ | 45.85^ab^ | 48.04^ab^ | 60.61^ab^ | 76.26^ab^ | 70.11^ab^ | 81.14^a^ | 61.34^ab^ | 78.81^a^ | 68.31^ab^ | 55.30^ab^ | 12.36 | <0.01 |
|  | LG |  | 74.57^ab^ | 55.99^abc^ | 36.31^c^ | 44.04^bc^ | 41.64^c^ | 59.28^abc^ | 74.15^ab^ | 71.62^ab^ | 75.83^ab^ | 57.36^abc^ | 80.40^a^ | 65.88^ab^ | 52.13^abc^ | 10.73 | <0.01 |
| - | | CP | 11.66^c^ | 39.54^ab^ | 32.07^b^ | 10.89^c^ | 33.28^b^ | 46.36^a^ | 13.33^c^ | 35.22^b^ | 10.62^c^ | 12.31^c^ | 53.24^a^ | 14.81^c^ | 49.19^a^ | 4.56^c^ | <0.01 |

^1^NRC, National Research Council Nutrient Requirements of Dogs and Cats (2006); AAFCO, Association of American Feed Control Officials (2016); FEDIAF, European Pet Food Industry Federation Nutritional Guidelines for Complete and Complementary Pet Foods for Cats and Dogs (2018).
^2^DIAAS-like, digestible indispensable amino acid score-like values; CP, crude protein content (%, dry matter basis).
^3^AM, adult maintenance; EG, early growth (NRC, 4-14 weeks of age; FEDIAF, less than 14 weeks of age); LG, late growth (NRC and FEDAF, greater than 14 weeks of age), GR, growth and reproduction (AAFCO only).
^a,b,c^ Values in a row with different superscript are significantly different (*P* ≤ 0.05).

**Supplementary Table 3.** Least-squares means (± SEM) for digestible indispensable amino acid scores (DIAAS)-like values and crude protein content (CP, % dry matter basis) of plant (plant protein product, processed grain by-product, grain product) ingredients and of animal (animal protein product) and plant (plant protein product) ingredients categorized based on collective AAFCO Official Common and Usual Names and Definitions of Feed Ingredients as determined using NRC, AAFCO, or FEDIAF^1^ amino acid requirements or recommendations at each discrete life stage as a reference pattern.

| IAA reference pattern | | Variable^2^ | Collective AAFCO classification | | | | SEM | *P*-value |
| --- | --- | --- | --- | --- | --- | --- | --- | --- |
|  |  |  | Animal protein product | Plant protein product | Processed grain by-product | Grain product |  |  |
| NRC | AM^3^ | DIAAS-like | 35.50 | 31.92^b^ | 38.57^ab^ | 42.80^a^ | 3.21 | <0.01 |
|  | EG |  | 63.61 | 57.99 | 59.32 | 62.58 | 6.22 | 0.63 |
|  | LG |  | 63.15 | 59.91 | 60.09 | 63.41 | 6.26 | 0.86 |
| AAFCO | AM | DIAAS-like | 61.61 | 54.40^b^ | 61.00^ab^ | 68.49^a^ | 5.73 | 0.04 |
|  | GR |  | 54.98 | 54.43 | 49.25 | 52.77 | 4.87 | 0.70 |
| FEDIAF | AM | DIAAS-like | 54.15 | 48.03^b^ | 58.08^ab^ | 63.37^a^ | 4.91 | <0.01 |
|  | EG |  | 69.56 | 64.52 | 66.12 | 69.63 | 6.84 | 0.73 |
|  | LG |  | 64.22 | 65.15 | 65.94 | 67.39 | 6.38 | 0.95 |
| - | | CP | 62.99^*^ | 49.24^a^ | 21.47^b^ | 12.48^c^ | 3.02 | <0.01 |

^1^NRC, National Research Council Nutrient Requirements of Dogs and Cats (2006); AAFCO, Association of American Feed Control Officials (2016); FEDIAF, European Pet Food Industry Federation Nutritional Guidelines for Complete and Complementary Pet Foods for Cats and Dogs (2018).
^2^DIAAS-like, digestible indispensable amino acid score-like values; CP, crude protein content (%, dry matter basis).
^3^AM, adult maintenance; EG, early growth (NRC, 4-14 weeks of age; FEDIAF, less than 14 weeks of age); LG, late growth (NRC and FEDAF, greater than 14 weeks of age), GR, growth and reproduction (AAFCO only).
^a,b,c^ Values in a row (plant ingredients only; plant protein products, processed grain by-products; grain products) with different superscript are significantly different (*P* ≤ 0.05).
^*^Animal protein product values are significantly different (*P* ≤ 0.05) than the plant protein products in the same row.
